# Diagnostic signature, subtype classification, and immune infiltration of key m6A regulators in osteomyelitis patients

**DOI:** 10.3389/fgene.2022.1044264

**Published:** 2022-12-05

**Authors:** Xiangwen Shi, Haonan Ni, Yipeng Wu, Minzheng Guo, Bin Wang, Yue Zhang, Bihuan Zhang, Yongqing Xu

**Affiliations:** ^1^ School of Medicine, Kunming Medical University, Kunming, China; ^2^ Department of Orthopedic Surgery, 920th Hospital of Joint Logistics Support Force, Kunming, China; ^3^ Laboratory of Clinical Medical Center, Yunnan Traumatology and Orthopedics, Kunming, China

**Keywords:** N6-methyladenosine (m6A), osteomyelitis, diagnosis, immune infiltration, subtype

## Abstract

**Background:** As a recurrent inflammatory bone disease, the treatment of osteomyelitis is always a tricky problem in orthopaedics. N6-methyladenosine (m6A) regulators play significant roles in immune and inflammatory responses. Nevertheless, the function of m6A modification in osteomyelitis remains unclear.

**Methods:** Based on the key m6A regulators selected by the GSE16129 dataset, a nomogram model was established to predict the incidence of osteomyelitis by using the random forest (RF) method. Through unsupervised clustering, osteomyelitis patients were divided into two m6A subtypes, and the immune infiltration of these subtypes was further evaluated. Validating the accuracy of the diagnostic model for osteomyelitis and the consistency of clustering based on the GSE30119 dataset.

**Results:** 3 writers of Methyltransferase-like 3 (METTL3), RNA-binding motif protein 15B (RBM15B) and Casitas B-lineage proto-oncogene like 1 (CBLL1) and three readers of YT521-B homology domain-containing protein 1 (YTHDC1), YT521-B homology domain-containing family 3 (YTHDF2) and Leucine-rich PPR motif-containing protein (LRPPRC) were identified by difference analysis, and their Mean Decrease Gini (MDG) scores were all greater than 10. Based on these 6 significant m6A regulators, a nomogram model was developed to predict the incidence of osteomyelitis, and the fitting curve indicated a high degree of fit in both the test and validation groups. Two m6A subtypes (cluster A and cluster B) were identified by the unsupervised clustering method, and there were significant differences in m6A scores and the abundance of immune infiltration between the two m6A subtypes. Among them, two m6A regulators (METTL3 and LRPPRC) were closely related to immune infiltration in patients with osteomyelitis.

**Conclusion:** m6A regulators play key roles in the molecular subtypes and immune response of osteomyelitis, which may provide assistance for personalized immunotherapy in patients with osteomyelitis.

## Introduction

Osteomyelitis is a devastating bone disease usually caused by bacterial, fungal and mycobacterial infections. Acute and chronic osteomyelitis both cause gradual deterioration of bone, as well as a severe inflammatory response, even invading the surrounding tissues outside the bone marrow cavity (Ma et al., 2021a). The incidence of posttraumatic osteomyelitis varies from 2% to 50% (Zalavras, 2017). In addition, diabetic ulcers due to vascular insufficiency are a common risk factor for osteomyelitis. The incidence of osteomyelitis is on the rise due to the increasing prevalence of diabetic foot infection. More than one-third of patients with diabetic foot infections are reported to be accompanied by osteomyelitis of the foot (Berendt et al., 2008). It is difficult to cure osteomyelitis, and even surgical intervention has an 8% mortality rate (Noskin et al., 2005).

The key to successful treatment of osteomyelitis is early diagnosis. However, the wide variation in clinical presentations of osteomyelitis makes accurate diagnosis of osteomyelitis challenging. The gold standard for the diagnosis of osteomyelitis includes bone histopathological examination and bacterial culture, which should be combined with imaging examination, laboratory test and physical examination (Hogan et al., 2013). The indicators of preliminary evaluation included erythrocyte sedimentation rate (ESR), C-reactive protein (CRP), and blood culture. Although CRP is elevated earlier than ESR and can be used to detect acute osteomyelitis, it is nonspecific for osteomyelitis (Unkila-Kallio et al., 1994; Peltola and Pääkkönen, 2014). Even when bacteria are cultured, 40% of osteomyelitis patients show negative results (Scott et al., 1990). In addition, early osteomyelitis is also difficult to diagnose with imaging, and the positive X-ray rate of patients with osteomyelitis for 2 weeks has been reported to be less than 20% (Pasquet et al., 2015). Therefore, no specific markers or methods have been identified for the diagnosis of osteomyelitis.

N6-methyladenosine (m6A) is the most prevalent chemical modification throughout the eukaryotic population, occurring in messenger RNAs (mRNAs), transfer RNAs (tRNAs) and ribosomal RNAs (rRNAs) (Linder et al., 2015; Xiao et al., 2016; Wang et al., 2020). The m6A modification process is mainly regulated by a combination of methylation recognition proteins (readers), m6A methyltransferases (writers), and m6A demethylases (erasers), which perform the functions of recognition, installation, and removal of methylation, respectively (Shi et al., 2019; Huang et al., 2021). Due to its reversible modifications that can affect the transcription and translation of mRNA profiles, which in turn regulate various fundamental cellular processes such as differentiation, metabolism, inflammation and immunity (Zhao et al., 2020; Zhu et al., 2020; Liu et al., 2022), it has begun to receive widespread attention from researchers.

Recently, m6A modifications have been found to be involved in the development of various inflammatory diseases, including skeletal diseases, neurodegenerative diseases, cardiac diseases, metabolic diseases, and cancer (Chokkalla et al., 2019; Zong et al., 2019; Zhang et al., 2020a). Zhang et al. (2019) found that METTL3 knockdown inhibited osteoblast differentiation and mineralization *via* the Smad signaling pathway under LPS-stimulated inflammatory conditions. In addition, METTL3 depletion also mediates the inflammatory response of osteoblasts through the MAPK signaling pathway, as evidenced by the production of inflammatory cytokines such as IL6 and IL12. However, the relationship and regulatory mechanisms between osteomyelitis and m6A modifications remain unclear. In this study, we used a random forest (RF) approach to establish a diagnostic model of osteomyelitis, and further two subtypes (m6A clusters and gene clusters) were identified using unsupervised clustering in patients with osteomyelitis. This study is designed to assist in the diagnosis of osteomyelitis and personalized immunotherapy based on different molecular subtypes.

## Material and methods

### Data collection and processing

Two gene expression datasets (GSE16129 and GSE30119) containing osteomyelitis and healthy samples were selected for this study and both were downloaded from the GEO database website (https://www.ncbi.nlm.nih.gov/geo/). GSE16129 datasets came from three platforms (GPL96, GPL97 and GPL6106), while GSE30119 datasets came from the GPL6947 platform. The gene expression data were merged using the “Perl” language (http://www.perl.org/) and normalized using the “sva” and “limma” packages of R software (Ritchie et al., 2015). As the merged microarray data come from three different platforms, the ComBat method was applied to correct the data in batches to eliminate the batch effects caused by multiple factors (Larsen et al., 2014; Müller et al., 2016; Zhang et al., 2020b). 97 osteomyelitis samples and 29 healthy samples from the GSE16129 dataset served as the test group, and 39 osteomyelitis samples and 44 healthy samples from the GSE30119 dataset served as the validation group. Data with large gene expression were changed with log2.

### Differential analysis of m6A regulators expression in osteomyelitis patients and healthy samples

26 m6A regulators were selected as references, including 15 readers, 9 writers and 2 erasers (Table 1) (Boulias et al., 2019; Zhang et al., 2020a). Based on these genes, the expression data of m6A regulators from 97 patients with osteomyelitis and 29 healthy samples in the text group were extracted by “limma” package, and the DEGs were screened by differential analysis between two groups. The results were visualized in the form of heatmap and boxplot with “pheatmap” and “ggpubr” packages (Zhao et al., 2014). The filter criteria were set to |logFC| value >1 and *p*-value < 0.05. In addition, the corresponding chromosome characteristics of m6A regulators, such as name, starting position and ending position, were obtained by consulting the literature (Dai et al., 2021). The locations of m6A regulators on chromosomes were extracted using the “Perl” program and were presented as a circle diagram using “RCirco” package (Zhang et al., 2013).

**TABLE 1:** The characteristics of 26 m6A regulators.

Type	Gene	Number
Writers	METTL3/METTL14/METTL16/WTAP/VIRMA/ZC3H13/RBM15/RBM15B/CBLL1	9
Readers	YTHDC1/YTHDC2/YTHDF1/YTHDF2/YTHDF3/HNRNPC/FMR1/LRPPRC/HNRNPA2B1/IGFBP1/IGFBP2/IGFBP3/RBMX/ELAVL1/IGF2BP1	15
Erasers	FTO/ALKBH5	2

### Diagnostic signature of osteomyelitis based on m6A regulators

Machine learning algorithms such as random forest (RF) and support vector machine (SVM) have been used for identifying and classifying key features of diseases, detecting hard-to-discern features in complex datasets, and analyzing gene expression data (Huang et al., 2018). RF is an integrated classifier consisting of many decision trees that exhibits robust performance by injecting randomness into the training of the trees to prevent overfitting of the data (Polan et al., 2016). SVM is also very effective in identifying complex datasets and is often used to identify cancer subtypes (Abinash and Vasudevan, 2022).

To better identify and screen important m6A regulators, two models were built by RF and SVM algorithms based on the text group. The boxplot of residual error, receiver operating characteristic (ROC) and reverse cumulative distribution plot of residual error were constructed to compare the accuracy of the two models. The smaller the residual error, the higher the accuracy of the models. After determining the model, the m6A regulators were scored according to their importance distribution, expressed as the Mean Decrease Gini (MDG). A higher value of a gene means that the gene is more important in this model. Genes with MDG values >10 were ultimately screened out. The “rms” package was then used to construct a nomogram model to predict the incidence of osteomyelitis. Calibration curves and decision curve analysis (DCA) were used in the analysis to assess the fit of the model, and a clinical impact curve was drawn to evaluate whether the model was beneficial to patients (Iasonos et al., 2008). Finally, the diagnostic model for osteomyelitis was validated based on the GSE30119 dataset.

### Identification of molecular subtypes based on significant m6A regulators

In consensus clustering, the K-means algorithm is run multiple times to obtain the input partition, and the common matrix is calculated based on the partition result. The ultimate goal is to detect sample subtypes with similar characteristics (Brière et al., 2021). The “ConsensusClusterPlus” package of R software was used to cluster osteomyelitis samples with m6A regulators to create subtypes with differential characteristics (Seiler et al., 2010; Yu et al., 2012). The most appropriate number of clusters was screened based on the consistency heatmap, consistency scores, cumulative distribution function (CDF) and CDF delta area curve. Specifically, the value of K corresponding to when the CDF reaches its approximate maximum value is the best grouping result (Wilkerson and Hayes, 2010). The maximum value of the number of clusters K was set to 10. To test the accuracy of clusters, the results of consensus clustering were validated based on the GSE30119 dataset and principal component analysis (PCA) was performed on the classified samples to determine whether the groupings accurately reflected the characteristics of osteomyelitis patients (David and Jacobs, 2014). Furthermore, the expression of m6A regulators was further analyzed to determine whether it differed significantly between molecular subtypes.

## Correlation analysis between m6A subtypes and immune cell infiltration

We further used single-sample gene set enrichment analysis (ssGSEA) in the “gsva” package to combine the 23 immune gene datasets with “high-low discriminant analysis” to calculate the immune infiltration score for each sample (Xiao et al., 2020). The boxplot was used to indicate whether the abundance of immune cells differed between m6A subtypes. The correlation between m6A regulators and immune cell infiltration was represented by a heatmap, and the top 2 m6A regulators with the strongest correlation were screened. The samples were divided into low and high expression groups based on the median expression value of each gene, and boxplots were used to observe whether there was a significant difference in the abundance of immune cells between the high and low expression groups.

## Correlation analysis between m6A subtypes and clinical characteristics

To test the relationship between the two m6A subtypes of osteomyelitis and clinical characteristics, information on the age and sex of osteomyelitis patients in the test and validation groups was extracted to further compare whether there were significant differences in age and sex between the two subtypes, represented in the form of boxplots and histograms. Furthermore, the results of clinical correlation were validated based on the GSE30119 dataset.

### GO functional annotation and KEGG pathway analysis based on DEGs between the two m6A subgroups

To explore the molecular mechanisms of m6A regulators, GO and KEGG enrichment analyses were performed using the “ClusterProfile” and “enrichplot” packages (The Gene Ontology, 2006; Kanehisa et al., 2012). In the GO enrichment analysis, the top 10 molecular functions (MF), cellular components (CC) and biological processes (BP) related to DEGs were screened, while the KEGG analysis focused on the biological pathways involved in DEGs.

### Identification of molecular subtypes based on the DEGs of two m6A subgroups

Based on the DEGs of the two m6A subtypes, consensus clustering analysis was performed using the “ClusterProfile” package. A consistency heatmap and CDF were used to determine the optimal number of clusters. Clustering conditions were set as follows: maxK = 8, reps = 50, pFeature = 1, pItem = 0.8, clusterAlg = “km”, distance = “euclidean”, and seed = 123,456.

### Correlation analysis between gene subgroups and immune cell infiltration

To explore the relationship between the 23 immune cells and the gene cluster, a boxplot was used to visualize whether the abundance of immune cells was significantly different in each molecular subtype. The expression of m6A regulators was also visualized using boxplots to determine whether it was different between gene subtypes.

### Differential analysis of m6A scores between the m6A subgroups or gene subgroups

Based on the expression of m6A regulators in each sample, the m6A score of each sample was obtained using PCA. Next, the “ggpubr” package was used to assess whether there were significant differences in m6A scores between the two m6A molecular subtypes or gene molecular subtypes. A Sankey diagram was drawn using the “ggalluvial” package to compare the similarity between m6A molecular subtypes and gene molecular subtypes. Finally, differences in m6A scores were validated based on the GSE30119 dataset.

### Differential analysis of collagen and interleukin-related genes between the m6A subgroups or gene subgroups

To further understand the relationship between m6A modification and the occurrence and development of osteomyelitis, we screened collagen and interleukin-related genes, which are involved in osteogenic differentiation and osteomyelitis inflammation, respectively. Boxplots were used to observe whether there were significant differences in the expression of these genes between the two m6A subtypes or gene subtypes. The expression of differences in collagen and interleukin-related genes were validated based on the GSE30119 dataset.

### Establishment of osteomyelitis model in rats

Twenty 8-week-old male SD rats weighing approximately 300 g were purchased from Kunming Animal Institute (Kunming, China), of which 10 were used as the experimental group and 10 as the control group. After anesthesia with intravenous sodium pentobarbital, the proximal third of the right tibia was exposed, and the medullary cavity was drilled approximately 1.5 mm and injected with 5 μl of 1 × 10^6^ *S. aureus*. The control group was injected with sterile phosphate buffer solution. The specimen was sealed with bone wax after implantation of the 5 mm × 1 mm steel needle. The specific procedure of the experimental model was based on the method of Vergidis et al. (2015). All experimental procedures were reviewed and approved by the 920th Hospital of Joint Logistics Support Force Ethics Review Committee (2022–025–01).

### Quantitative real-time PCR

Tibial tissues from the 10 rat models of osteomyelitis and 10 control groups were collected, and total RNA was extracted from tibial tissues using TRIzol reagent (Ambion Inc., Austin, TX), followed by reverse transcription of RNA to cDNA using SweScript RT I First Strand cDNA Synthesis Kit (Service Bio, Guangzhou, China) to reverse transcribe RNA to cDNA. Quantitative real-time PCR was performed using Universal Blue SYBR Green qPCR Master Mix (Service Bio, Guangzhou, China), and the level of GAPDH was used as an internal control. Relative expression was calculated according to the comparative Ct (2^−ΔΔCT^) method (Livak and Schmittgen, 2001). The sequences of the primers used are shown in Supplementary Table S1.

### Immunohistochemistry

Right tibial tissues from the experimental group and the control group in the same location were taken and immunohistochemically stained using 4% paraformaldehyde fixation and paraffin embedding. Following paraffin removal, experimental tissues were incubated with primary antibodies against METTL3, YTHDC1, YTHDF2, RBM15B, LRPPRC and CBLL1 overnight at 4°C and control tissues were incubated with sterile phosphate-buffered saline (PBS; Service Bio, Guangzhou, China). Representative slices were incubated with biotinylated secondary antibodies (Service Bio, Guangzhou, China) at 22°C (Ramos-Vara, 2005). After the samples were sectioned, the staining differences of 6 significant m6A regulators in tibial tissues were evaluated under the microscope.

### Statistical analysis

The Kruskal‒Wallis test was used to compare the differences between groups, with *p* < 0.05 indicating statistical significance. All statistical analyses were performed using R software (version 4.1.3) and the corresponding program package (http://www.bioconductor.org/).

## Results

### Expression and specific characteristics of m6A regulators in osteomyelitis patients and healthy samples

**FIGURE 1:**
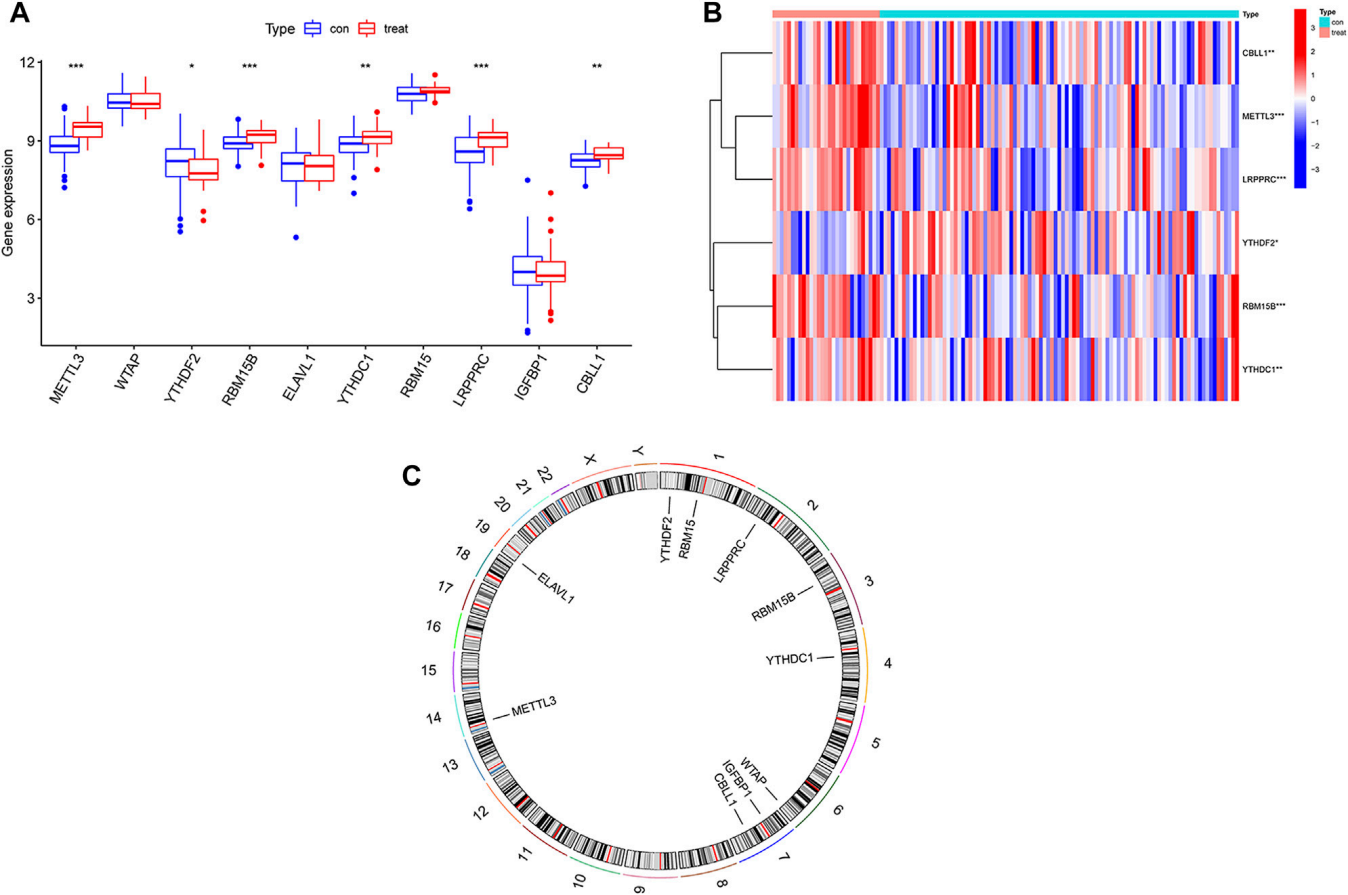
Expression of m6A regulators in osteomyelitis samples and healthy samples. **(A)** Expression of all 10 m6A regulators in osteomyelitis samples and normal samples. **(B)** Heatmap of 6 significant m6A regulators between the two groups. **(C)** Chromosomal characteristics of all 10 m6A regulators.

The test group consisted of 97 osteomyelitis samples and 29 healthy samples, including 71 males and 55 females, with an average age of 7.4 ± 4.7 years, and the validation group consisted of 39 osteomyelitis samples and 44 healthy samples, including 41 males and 42 females, with an average age of 7.2 ± 4.3 years. Based on the 26 m6A regulators used as reference, 10 m6A regulators were identified in all samples and then screened for 6 m6A regulators after differential expression analysis (Figures 1A,B). The 6 m6A regulators include 3 writers of Methyltransferase-like 3 (METTL3), RNA-binding motif protein 15B (RBM15B) and Casitas B-lineage proto-oncogene like 1 (CBLL1) and three readers of YT521-B homology domain-containing protein 1 (YTHDC1), YT521-B homology domain-containing family 3 (YTHDF2) and Leucine-rich PPR motif-containing protein (LRPPRC). In addition, we observed the chromosome characteristics of m6A regulators in the form of a circle diagram and found that m6A regulators associated with osteomyelitis were mainly distributed on chromosomes 1, 2, 3, 4, 6, 7, 14 and 19 (Figure 1C). Specific characteristics of chromosomes, including starting position and ending position, are shown in Table 2.

**TABLE 2:** Specific characteristics of m6A regulators on chromosomes.

Gene	Chromosome	Starting position	Ending position
METTL3	Chromosome 14	21498133	21511375
WTAP	Chromosome 6	159725585	159756319
RBM15	Chromosome 1	110338506	110346681
RBM15B	Chromosome 3	51391268	51397908
CBLL1	Chromosome 7	107743697	107761667
YTHDC1	Chromosome 4	68310387	68350089
YTHDF2	Chromosome 1	28736621	28769775
LRPPRC	Chromosome 2	43886508	43996005
IGFBP1	Chromosome 7	45888357	45893668
ELAVL1	Chromosome 19	7958579	8445041

### Diagnostic signature for osteomyelitis based on m6A regulators

**FIGURE 2:**
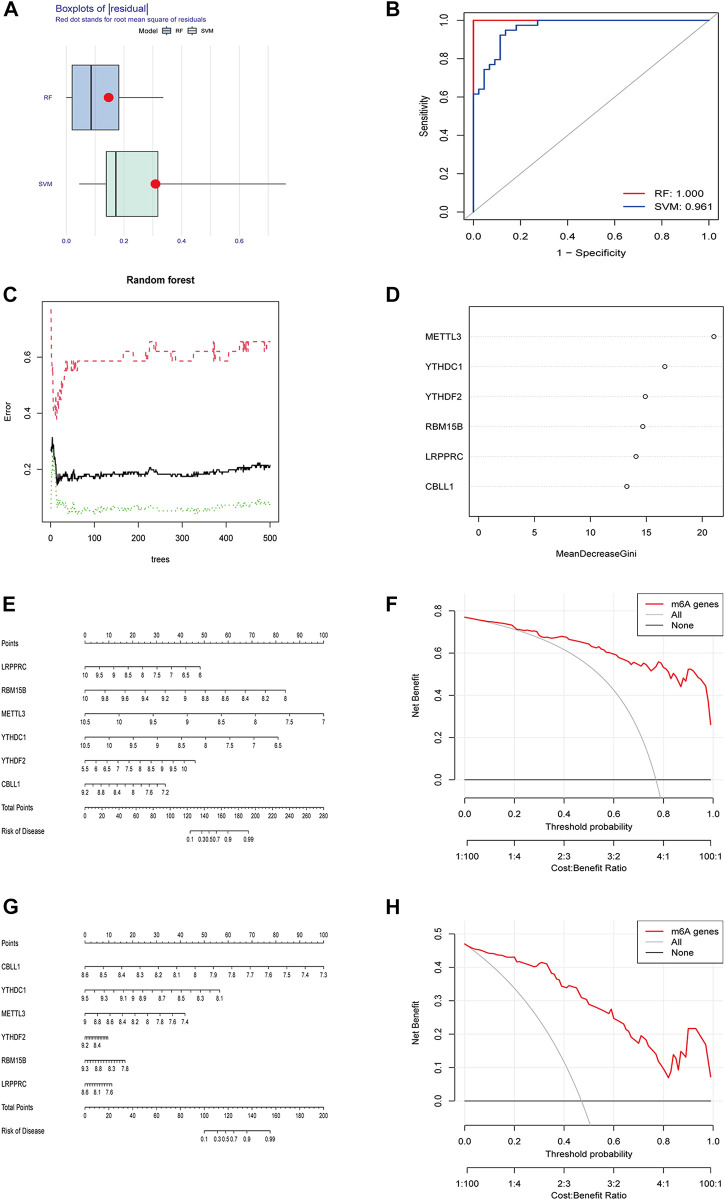
Building and validating of diagnostic models based on RF and SVM machine learning methods. **(A)** Boxplot of residuals. **(B)** ROC curves for both models. **(C)** Construction of random forest model. **(D)** MDG value of 6 significant m6A-related DEGs. **(E)** Nomogram of the model based on the GSE16129 dataset. **(F)** Calibration curve of the text model. **(G)** Nomogram of the model based on the GSE30119 dataset. **(H)** Calibration curve of the validation model. RF, random forest; SVM, support vector machine; ROC, receiver operating characteristic; MDG, Mean Decrease Gini; DCA, Decision Curve Analysis.

RF and SVM were used to construct diagnostic signatures. The boxplot and line plot revealed that the residual of RF was smaller than that of SVM (Figure 2A), and the AUC value of the model built by RF was larger than that of SVM (RF: 1.000 vs. SVM: 0.961) (Figure 2B). Therefore, RF model was selected as the best model to predict the incidence of osteomyelitis. The RF model was constructed based on the trees (Figure 2C), of which 6 significant m6A regulators (METTL3, YTHDC1, YTHDF2, RBM15B, LRPPRC and CBLL1) had MDG values greater than 10, and all of them were included in this RF model (Figure 2D). Furthermore, a nomogram model was established to predict the incidence of osteomyelitis by summing the scores for each gene (Figure 2E). In general, a higher total score represented a higher risk of osteomyelitis. The calibration curve and DCA curve suggested that the RF model fit well (Figure 2F). In the validation group, a nomogram model (Figure 2G) was established to predict the incidence of osteomyelitis and the calibration curve and DCA curve also suggested that the RF model fit well (Figure 2H).

## Results of molecular subtypes based on m6A regulators

**FIGURE 3:**
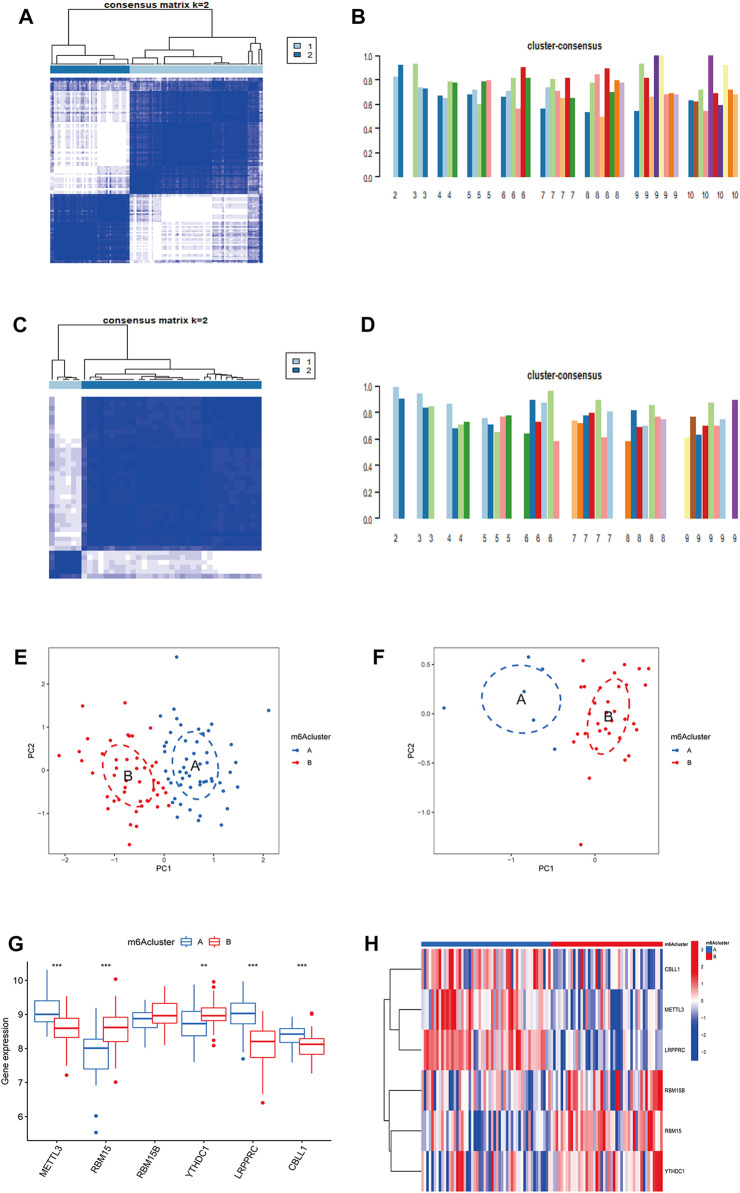
Molecular subgroups based on clustering analysis of m6A regulators. **(A)** Heatmap of 2 clusters (k = 2) based on m6A regulators from the GSE16129 dataset. **(B)** Consistency scores of all the text clusters (k = 2–10). **(C)** Heatmap of 2 clusters (k = 2) based on m6A regulators from the GSE30119 dataset. **(D)** Consistency scores of all the validation clusters (k = 2–9). **(E)** PCA analysis of the 2 clusters in the text group. **(F)** PCA analysis of the 2 clusters in the validation group. **(G)** Box diagram of differential expression of 6 significant m6A regulators in two m6A clusters from the text group. **(H)** Heatmap of differential expression of 6 significant m6A regulators in two m6A clusters from the text group.

Based on CDF, consistency score, CDF delta area curve and number of samples, two molecular subtypes of m6A were identified (cluster A and cluster B) and consistency score (k = 2) > 0.8 (Figures 3A,B). There were 45 samples in cluster A osteomyelitis and 52 samples in cluster B osteomyelitis. In the validation group, cluster A osteomyelitis included 6 samples and cluster B osteomyelitis included 33 samples (Figures 3C,D). The results of PCA suggested that cluster A and cluster B osteomyelitis were well distinguished by the expression of m6A regulators in both the text and validation groups (Figures 3E,F). Visualization of the results by boxplot and heatmap suggested that the 5 regulators were differentially expressed between the two m6A subtypes, with METTL3, CBLL1 and LRPPRC highly expressed in cluster A osteomyelitis, RBM15B and YTHDC1 highly expressed in cluster B osteomyelitis (Figures 3G,H). The different m6A clusters of the test (k = 1–10) and validation groups (k = 1–9) can be found in Supplementary Figure S1.

### Immune infiltration analysis of m6A subtypes

**FIGURE 4:**
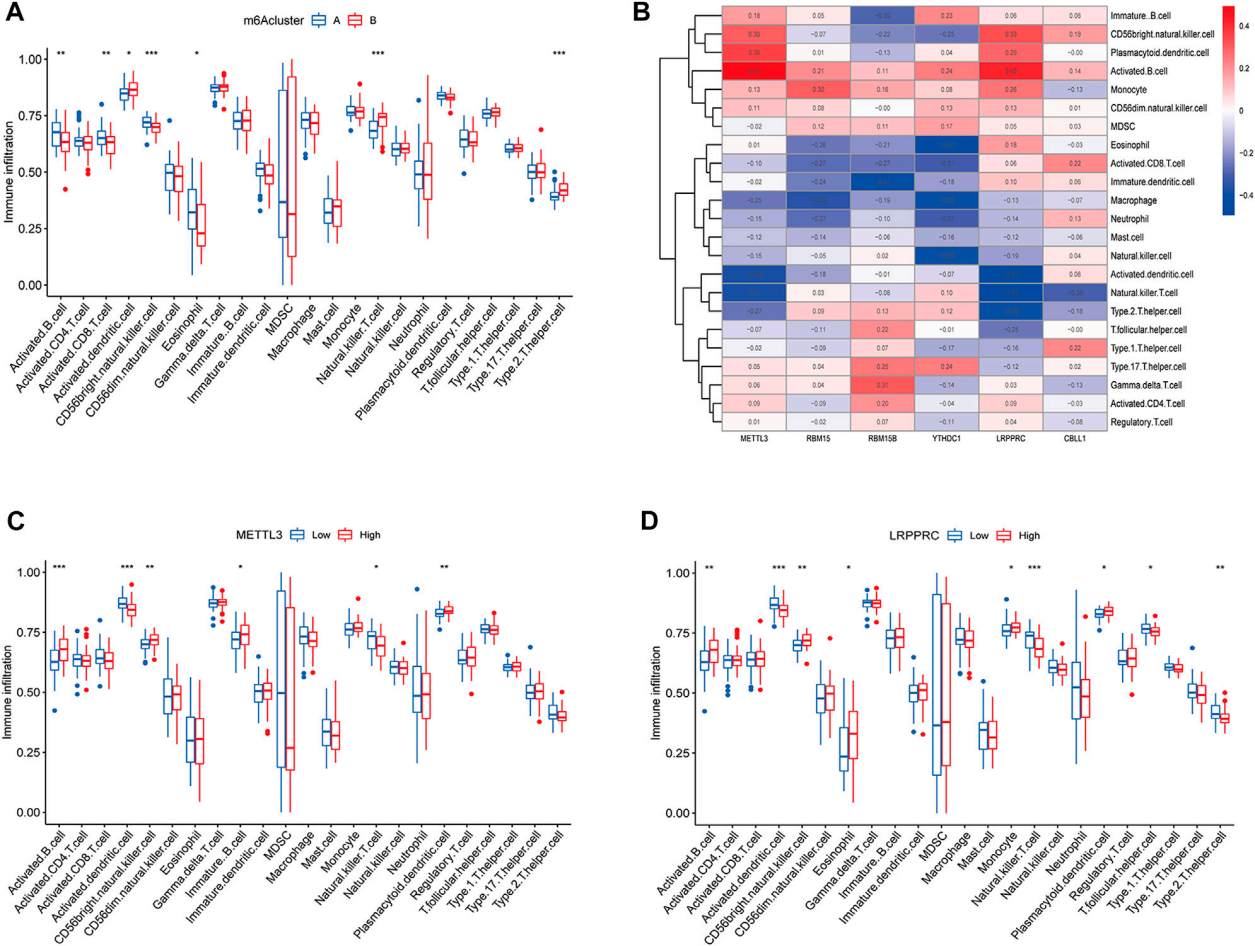
Immune cells infiltration analysis of m6A regulators. **(A)** Box diagram of immunocyte abundance in molecular subgroups of m6A regulators. **(B)** Heatmap of correlation between immunocyte abundance and m6A regulators expression. **(C)** Box diagram of comparison of immunocyte abundance in the METTL3 high- and low-expression groups. **(D)** Box diagram of comparison of immunocyte abundance in the LRPPRC high- and low-expression groups.

The results of ssGSEA analysis revealed that a total of 7 immune cells were significantly different in two m6A subtypes, among which activated B cells, activated CD8 T cells, CD56 bright natural killer cells, and eosinophils were highly abundant in cluster A osteomyelitis, while activated dendritic cells, natural killer cells, and type 2 T helper cells were highly abundant in cluster B osteomyelitis (Figure 4A). The correlation heatmap suggested that the METTL3 gene showed a strong positive correlation with activated B cells (r = 0.49), and a strong negative correlation with activated dendritic cells (r = −0.38) and natural killer T cells (r = −0.37). The LRPPRC gene also showed a strong negative correlation with natural killer T cells (r = −0.49) (Figure 4B). Therefore, METTL3 and LRPPRC were chosen to study their abundance of immune infiltration in the high and low expression groups (Figures 4C,D). In the METTL3 high-expressed group, activated B cells, CD56 bright natural killer cells, immature dendritic cells, and plasmacytoid dendritic cells were highly infiltrated, whereas in the low-expressed group, activated dendritic cells, and natural killer T cells were highly infiltrated in the low-expressed group. In the high LRPPRC expression group, activated B cells, CD56 bright natural killer cells, eosinophils, monocytes, and plasmacytoid dendritic cells were highly infiltrated, whereas in the low expression group, activated dendritic cells, natural killer T cells, T follicular helper cells, and type 2 T helper cells were highly infiltrated in the low-expressed group.

### Clinical correlation analysis of m6A subtypes

**FIGURE 5:**
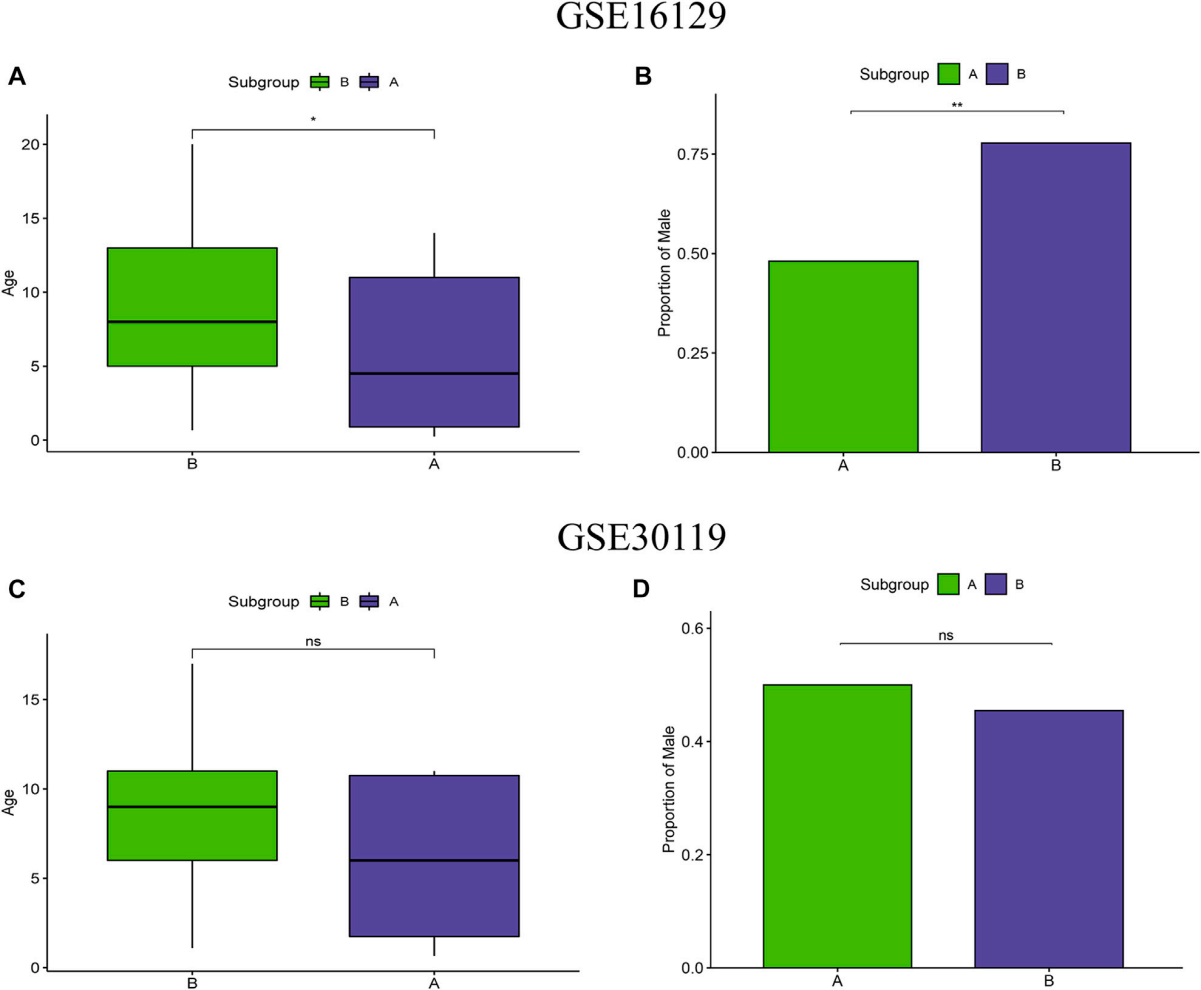
Clinical correlation analysis of two m6A clusters based on the GSE16129 and GSE30119 datasets. Differences in age **(A)** and proportion of male **(B)** between two m6A clusters based on the GSE16129 dataset. Differences in age **(C)** and proportion of male **(D)** between two m6A clusters based on the GSE30119 dataset.

To test the clinical correlation of m6A subtypes, we analyzed the relationship between the two m6A subtypes and clinical characteristics. In the m6A subtypes of the test group, age and proportion of male were significantly lower in subgroup A than in subgroup B (Figures 5A,B). However, there were no significant difference in age and proportion of male in the two m6A subtypes of the validation group (Figures 5C,D).

### Effects of GO functional annotation and KEGG pathway analysis

**FIGURE 6:**
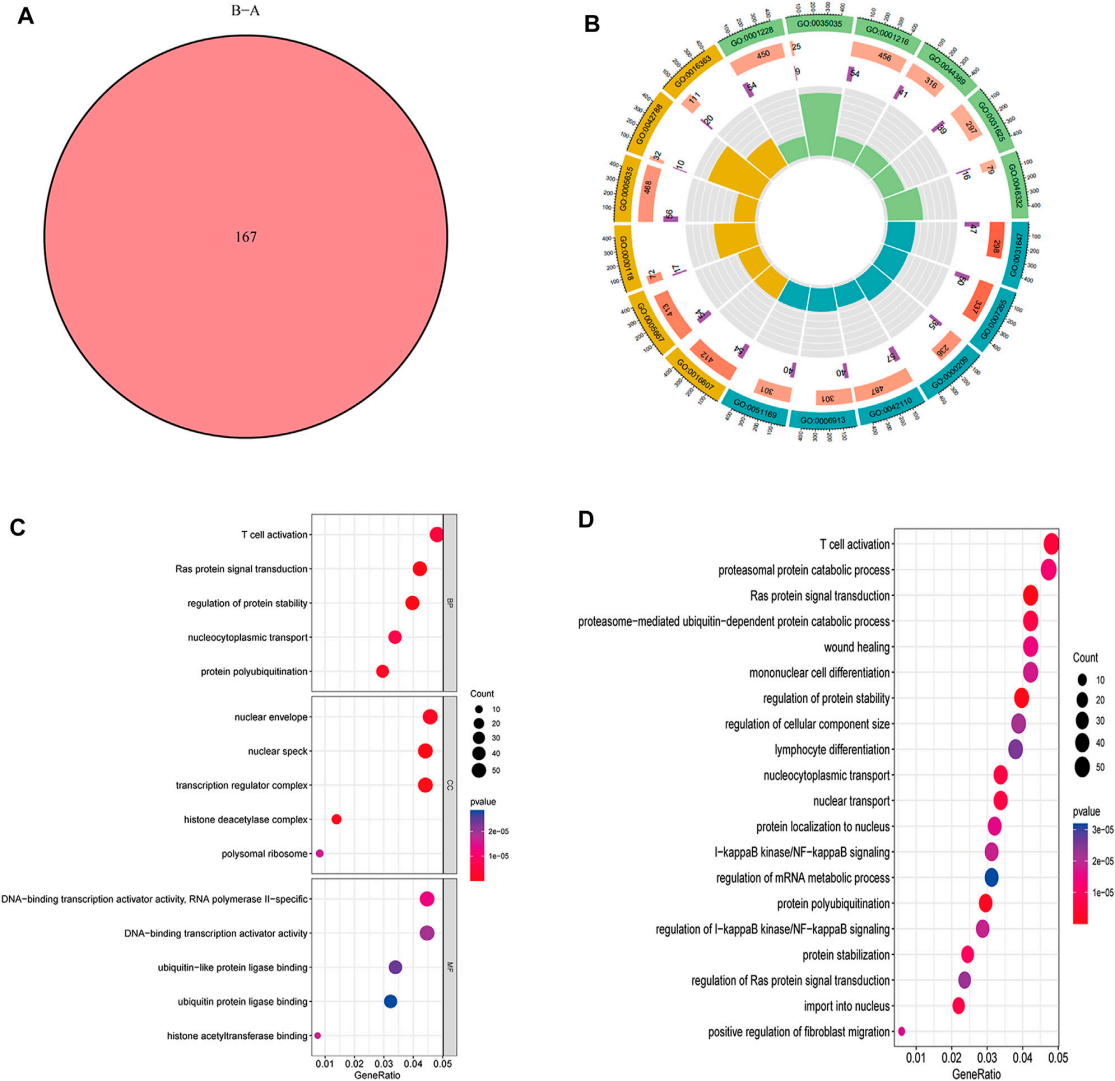
GO functional annotation and KEGG pathway analysis of DEGs based two m6A subgroups. **(A)** Venn diagram of DEGs. Circle **(B)** and bubble diagram **(C)** of GO functional annotation. **(D)** Top 20 KEGG pathway analysis of DEGs.

A total of 167 m6A-related DEGs were obtained from the two m6A subtypes using the “limma” and “VennDiagram” packages (Figure 6A). To understand the molecular functions and biological processes of the DEGs, GO annotation and KEGG pathway analysis were performed on DEGs from cluster A osteomyelitis and cluster B osteomyelitis (Figures 6B–D). In GO analysis, DEGs were mainly involved in biological processes (BP) such as T cell activation, Ras protein signal transduction, and regulation of protein stability; cellular components (CC) such as nuclear envelope, nuclear speck, and transcription regulator complex; and molecular functions (MF) such as RNA polymerase II−specific, DNA−binding transcription activator activity, and ubiquitin−like protein ligase binding. KEGG pathway analysis revealed that DEGs were mainly involved in T cell activation and proteasomal protein catabolic processes.

### Immune infiltration of gene subtypes

**FIGURE 7:**
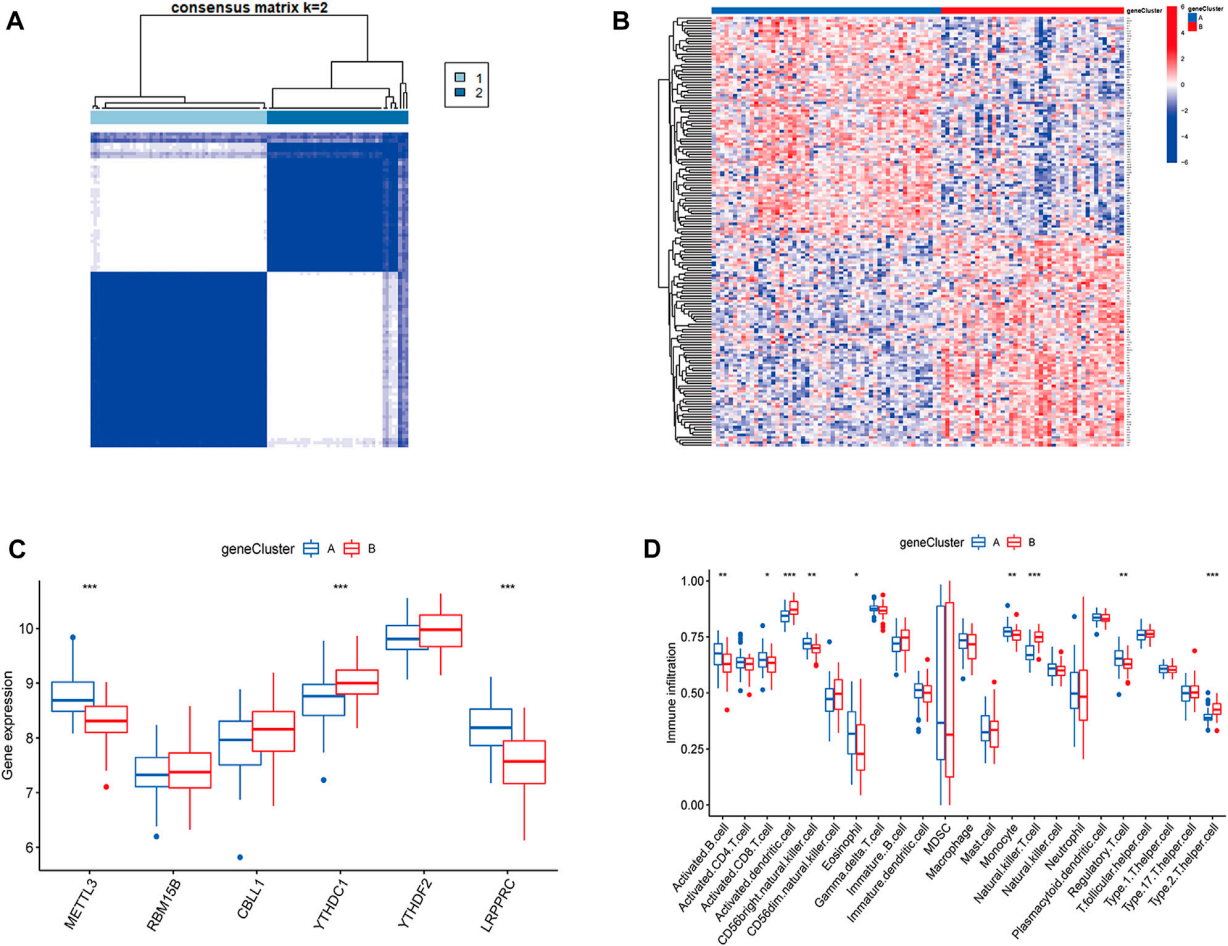
Molecular subgroups and immune cells infiltration analysis based on m6A-associated DEGs. **(A)** Clustered heatmap of 2 clusters (k = 2) based on m6A-associated DEGs. **(B)** Heatmap of typing for 167 DEGs. **(C)** Box diagram of m6A regulators between the two gene subgroups. **(D)** Box diagram of immunocyte abundance between the two gene subgroups.

To validate the m6A subtypes, two molecular subtypes were further confirmed based on 167 DEGs of the m6A subtypes (gene cluster A and gene cluster B) (Figures 7A,B). There were 54 cases of gene cluster A osteomyelitis and 43 cases of gene cluster B osteomyelitis. Among the 6 m6A regulators, METTL3 and LRPPRC were highly expressed in subgroup A osteomyelitis, whereas YTHDC1 was highly expressed in gene cluster B osteomyelitis (Figure 7C). In addition, the results of immune infiltration showed that 9 immune cells were significantly different between the two gene subtypes, in which activated B cells, activated CD8 T cells, CD56 bright natural killer cells, eosinophils, monocytes, and regulatory T cells were highly infiltrated in gene cluster A, while activated dendritic cells, natural killer T cells, and type 2 T helper cells were highly infiltrated in gene cluster B (Figure 7D).

### Differential analysis of m6A scores, collagen- and interleukin-related genes in the two typing methods

**FIGURE 8:**
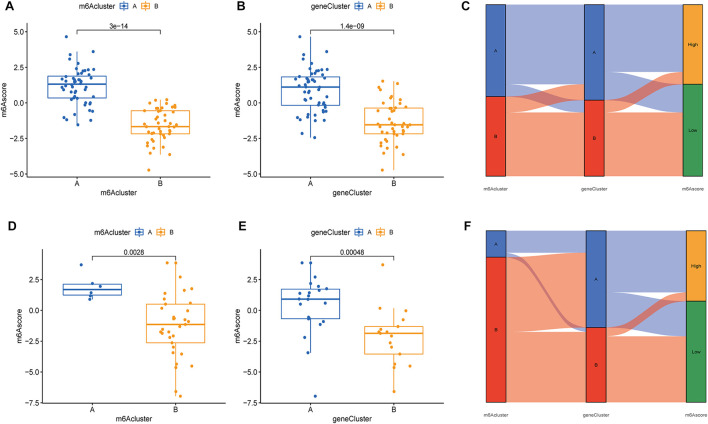
Differential analysis of m6A scores in the two typing methods based on the GSE16129 and GSE30119 datasets. Differential analysis of m6A scores in the two m6A subgroups **(A)** and two gene subgroups **(B)** based on the GSE16129. **(C)** A Sankey diagram was showed the relationship between m6A subgroups, m6A gene subgroups, and m6A scores based on the GSE16129. Differential analysis of m6A scores in the two m6A subgroups **(D)** and two gene subgroups **(E)** based on the GSE30119. **(F)** A Sankey diagram was showed the relationship between m6A subgroups, m6A gene subgroups, and m6A scores based on the GSE30119.

Based on the expression of m6A regulators, 97 samples from the test group and 83 samples from the validation group were scored using PCA analysis, and the score of each sample is shown in Supplementary Table S2. We then compared m6A scores in the two typing approaches, and the results showed that m6A scores in cluster B or gene cluster B were significantly higher than those in cluster A or gene cluster A (Figures 8A,B). The Sankey diagram suggested a high similarity between m6A subtypes and gene subtypes (Figure 8C). In the validation group, the m6A scores in cluster B or gene cluster B were also significantly higher than those in cluster A or gene cluster A (Figures 8D,E) and the Sankey diagram suggested a similarity between m6A subtypes and gene subtypes (Figure 8F).

**FIGURE 9:**
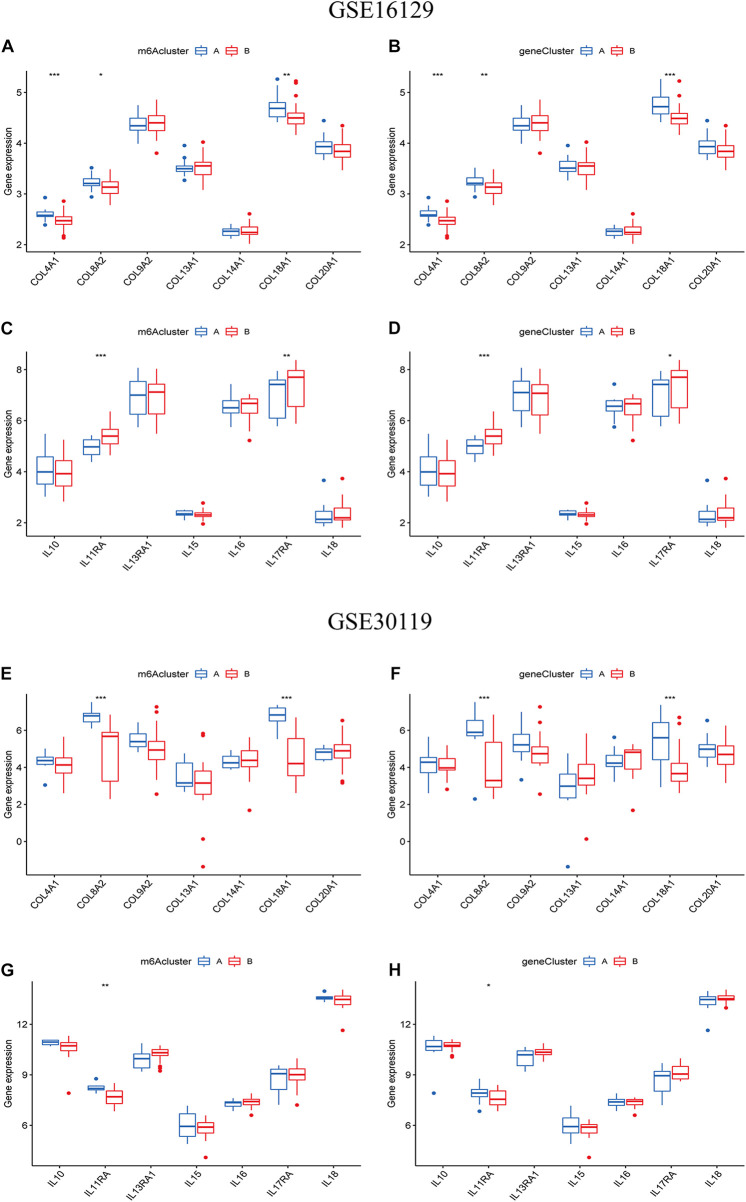
Differential analysis of collagen-related and interleukin-related genes in the two typing methods. Differential analysis of collagen-related genes between the two m6A **(A)** and two gene subgroups **(B)** based on the GSE16129. Differential analysis of interleukin-related genes between the two m6A **(C)** and two gene subgroups **(D)** based on the GSE16129. Differential analysis of collagen-related genes between the two m6A **(E)** and two gene subgroups **(F)** based on the GSE30119. Differential analysis of interleukin-related genes between the two m6A **(G)** and two gene subgroups **(H)** based on the GSE30119.

To further reveal the relationship between m6A subtypes and the development of osteomyelitis, we found that between the two m6A subtypes from the test group, the expression of 3 collagen-related genes was significantly different, with COL4A1, COL8A2 and COL18A1 both highly expressed in cluster A osteomyelitis. The expression of 2 interleukin-related genes was significantly different, with IL11RA and IL17RA highly expressed in cluster B osteomyelitis (Figures 9A,B). Between the two gene subtypes, the expression of 3 collagen-related genes was significantly different, among which COL4A1, COL8A2 and COL18A1 were all highly expressed in gene cluster A osteomyelitis. The expression of 2 interleukin-related genes were significantly different, among which IL11RA and IL17RA were highly expressed in gene cluster B osteomyelitis (Figures 9C, D). Similar to the results of the test group, the validation group exhibited a weak correlation with collagen- and interleukin-related genes. Between the two m6A subtypes from the validation group, the expression of 2 collagen-related genes was significantly different, with COL8A2 and COL18A1 both highly expressed in cluster A osteomyelitis. The expression of 1 interleukin-related gene was significantly different, with IL11RA being highly expressed in cluster A osteomyelitis (Figures 9E,F). Between the two gene subtypes, the expression of 2 collagen-related genes was significantly different, among which COL8A2 and COL18A1 were all highly expressed in gene cluster A osteomyelitis. The expression of 2 interleukin-related genes was significantly different, among which IL11RA was highly expressed in gene cluster A osteomyelitis (Figures 9G,H).

### Validation of m6A regulators in rat models of osteomyelitis

**FIGURE 10:**
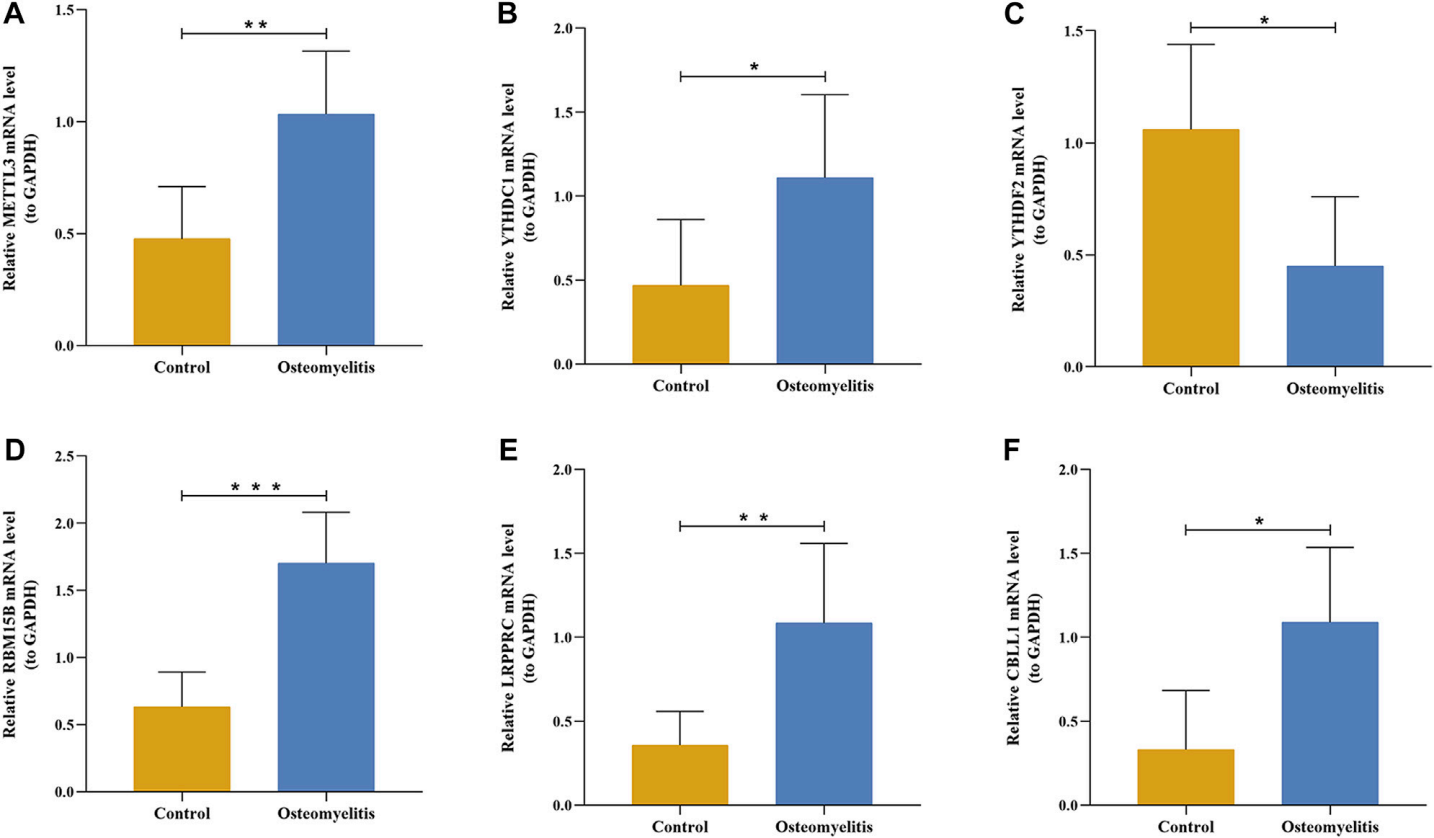
The mRNA expression levels of 6 significant m6A regulators in the focal bone tissue and healthy bone tissue of rat model of osteomyelitis. The mRNA expression levels of METTL3 **(A)**, YTHDC1 **(B)**, YTHDF2 **(C)**, RBM15B **(D)**, LRPPRC **(E)**, and CBLL1 **(F)** in focal bone tissues were significantly higher than that of healthy bone tissues.

**FIGURE 11:**
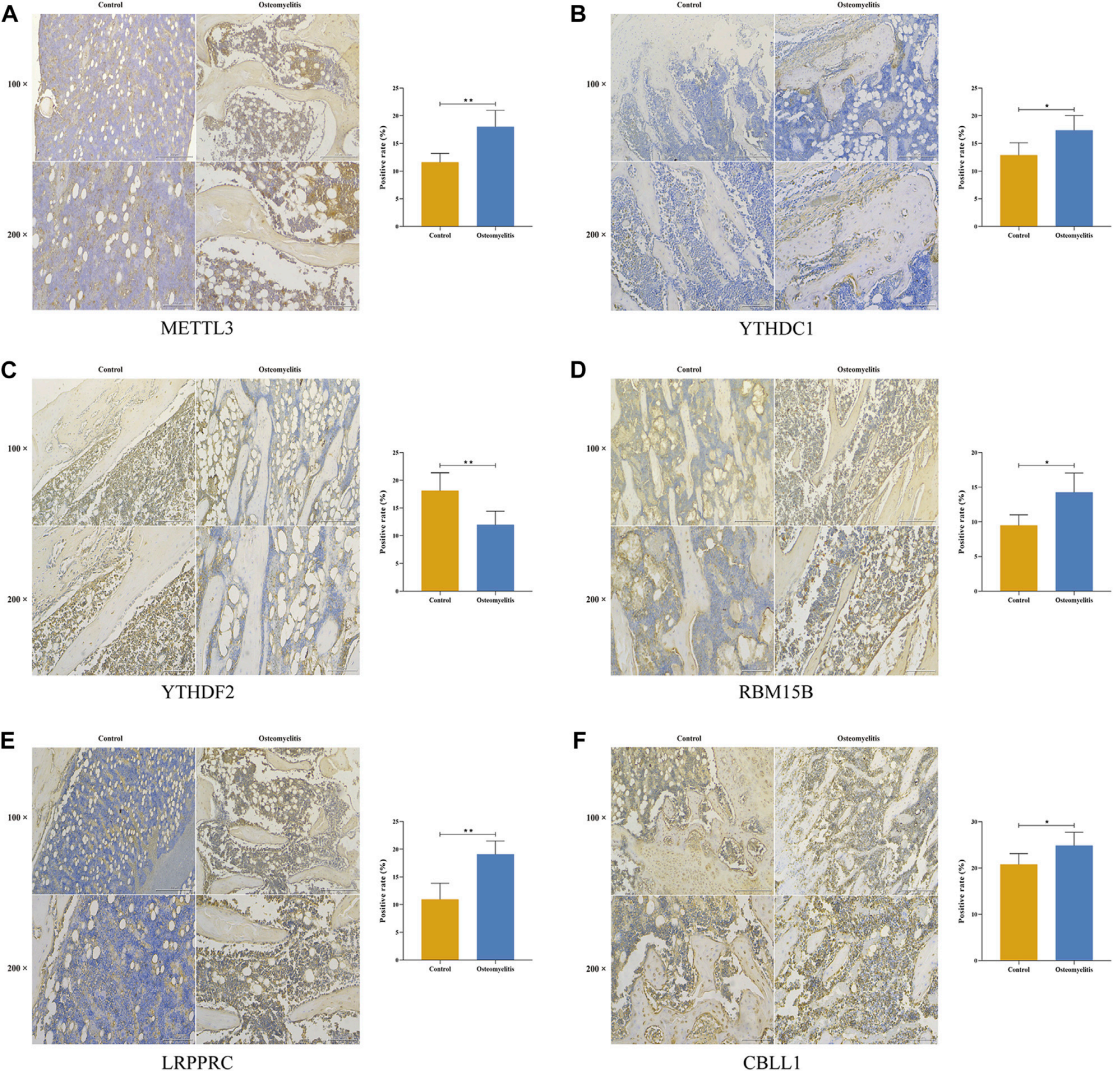
Immunohistochemistry of 6 significant m6A regulators in the focal bone tissue and healthy bone tissue of rat model of osteomyelitis. METTL3 **(A)**, YTHDC1 **(B)**, YTHDF2 **(C)**, RBM15B **(D)**, LRPPRC **(E)**, and CBLL1 **(F)** were abundantly expressed in focal bone tissues, and the positive rates were significantly higher than that of healthy bone tissues.

Based on the 6 significant m6A regulators with differential expression in the dataset, we further validated the expression of the 6 m6A regulators (METTL3, YTHDC1, YTHDF2, RBM15B, LRPPRC and CBLL1) in 10 osteomyelitis samples and 10 control samples by qRT‒PCR analysis. The qRT‒PCR results showed that the mRNA expression of METTL3, YTHDC1, RBM15B, LRPPRC and CBLL1 in osteomyelitis tissues was significantly increased, while that of YTHDF2 was significantly decreased (*p* < 0.05), which was consistent with the results of bioinformatics analysis (Figures 10A–F). To verify the effectiveness of our analysis, immunohistochemical staining was further performed on the focal bone tissue and healthy bone tissue of rat model of osteomyelitis (Figures 11A–F). The results suggested that METTL3, YTHDC1, RBM15B, LRPPRC and CBLL1 were highly expressed in the focal bone tissue of rats, while YTHDF2 was expressed at low levels. Compared with the healthy bone tissue, the positive rates of METTL3, YTHDC1, RBM15B, LRPPRC and CBLL1 in the focus bone tissue were higher, while YTHDF2 was lower (*p* < 0.05), which is consistent with the results of bioinformatics analysis and qRT‒PCR.

## Discussion

In this study, two datasets (GSE16129 and GSE30119) were collected, the GSE16129 dataset as the test group and the GSE30119 dataset as the validation group. A diagnostic model for osteomyelitis was established based on m6A regulators using a RF approach, and calibration curves and DCA plots demonstrated that the model fit well. In addition, we classified 97 osteomyelitis samples into two molecular subtypes based on the expression of 6 significant m6A regulators and 167 m6A-associated DEGs. The abundance of immune cell infiltration and genes involved in osteomyelitis were significantly different between the two m6A subtypes or gene subtypes. The accuracy of both the osteomyelitis diagnostic model and the m6A molecular subtypes were validated using the GSE30119 dataset. All these findings suggest that the diagnostic model and molecular subtypes were crucial to differentiate osteomyelitis. To our knowledge, this is the first study to comprehensively evaluate the functions of m6A regulators in the diagnosis and molecular subtypes of osteomyelitis.

The diagnostic model of osteomyelitis included 6 significant m6A-related DEGs, namely METTL3, YTHDC1, YTHDF2, RBM15B, LRPPRC and CBLL1, which all had importance scores greater than 10. A previous study found that METTL3 knockdown promoted the expression levels of the inflammatory cytokines IL6 and IL12, as well as the expression of osteogenic-related genes and osteoblast differentiation in an LPS-mediated inflammatory environment (Zhang et al., 2019). *In vitro*, overexpression of METTL3 can attenuate LPS-induced inflammatory responses in macrophages *via* the NF-κB pathway (Wang et al., 2019a). Silencing YTHDF2 exacerbated LPS-induced inflammatory response in RAW264.7 cells through activation of the NF-κB pathway (Yu et al., 2019). A recent study also demonstrated that METTL3 knockdown promoted proliferation and differentiation of osteoblasts under LPS-mediated inflammatory conditions and similar results were observed after YTHDF2 knockdown (Kong et al., 2022). These findings were similar to our results. We found that METTL3 was highly expressed and YTHDF2 was expressed at low levels in osteomyelitis samples and validated this finding in a rat model of osteomyelitis. It is suggested that METTL3 and YTHDF2 may be involved in promoting osteogenic differentiation and attenuating the inflammatory response in the inflammatory environment of osteomyelitis, making them potential therapeutic targets. 15 m6A regulators, including METTL3, YTHDC1, YTHDF2, RBM15B, LRPPRC and CBLL1, are involved in the pathological process of periodontitis (Zhang et al., 2021). Our study also came to similar conclusions, suggesting that these 6 m6A regulators may play a key regulatory role in the development of inflammatory diseases.

To investigate the role of m6A modification in disease, researchers were the first to conduct studies in the field of oncology (Liu et al., 2020; Zhao et al., 2021a; Li and Zhang, 2021). In the non-oncology field, studies based on consensus clustering analysis have been conducted to explore their regulatory mechanisms in periodontitis and acute myocardial infarction (Zhao et al., 2021b; Zhang et al., 2021). In the clustering analysis, two kinds of molecular subtypes were identified based on m6A regulators and gene clusters. In the m6A regulators-associated molecular subtypes, METTL3, CBLL1 and LRPPRC were significantly more highly expressed in subgroup A osteomyelitis than in subgroup B, while RBM15B and YTHDC1 expression were higher in subgroup A osteomyelitis. We obtained consistent findings in the gene subtypes. These results suggested that above 5 m6A regulators were important for distinguishing between subgroup A osteomyelitis and subgroup B osteomyelitis.

In addition, m6A modification has been proven to be involved in the regulation of the cellular immune response (Shriwas et al., 2020; Ma et al., 2021b; Liu et al., 2021). We found significant differences in the abundance of numerous immune cells in molecular subtypes based on either m6A clusters or gene clusters. In these subtypes, 7 immune cells were different in m6A subtypes, and 9 immune cells were different in gene subtypes. Specifically, there were significant differences between activated B cells, activated CD8 T cells, activated dendritic cells, CD56 bright natural killer cells, eosinophil, natural killer T cells, and type 2 T helper cells. We further identified METTL3 and LRPPRC as two m6A regulators with strong relevance to immune cell infiltration. 9 immune cell infiltration abundances were significantly different in METTL3, and 8 immune cells were significantly different in LRPPRC. Liu et al. found that METTL3 knockdown inhibited M1-type macrophages but promoted the polarization of M2-type macrophages (Liu et al., 2019). In our study, the proportion of monocyte infiltration was higher in the group with low METTL3 expression. On the other hand, METTL3 mediates the activation and maturation of dendritic cells. MTTTL3 knockout can reduce the production of viruses in B cells infected by viruses (Hesser et al., 2018; Wang et al., 2019b). In this study, we indicated that activated B cells had higher infiltration abundance in the group with high METTL3 expression, suggesting that METTL3 may play an important role in the immune response of B cells and be closely related to the immune regulation of osteomyelitis. However, there are still few studies on the regulatory role of LRPPRC in immune cells. In addition, the inflammatory response is mainly caused by immune cells and cytokines (Luo et al., 2021). To explore the role of inflammatory cytokines in osteomyelitis, we explored the expression of interleukin-related genes in molecular subtypes. The differences between IL11RA and IL17RA in the m6A cluster and gene cluster suggested that the two genes may be involved in osteomyelitis-mediated inflammation. Additionally, collagens and collagen-related genes have been shown to be involved in a variety of bone diseases (Myllyharju and Kivirikko, 2001; Marini and Blissett, 2013; Forlino and Marini, 2016), and we also analyzed the differential expression of collagen-related genes among molecular subtypes of osteomyelitis. The results suggested that both COL4A1 and COL18A1 were highly expressed in subgroup A osteomyelitis.

Our study has some certain limitations. Firstly, we tried to find additional datasets to validate the consistency of the osteomyelitis diagnostic model and clustering analysis, but no eligible datasets were found outside the GEO database. The GSE30119, as the only validation data set, leads to clustering results with unbalanced sample allocation and future large sample datasets for verification are necessary. Secondly, this study analyzed the relationship between m6A regulators and immune cell infiltration and briefly validated the expression of key m6A regulators in rat model of osteomyelitis, but its specific regulatory mechanism in osteomyelitis-mediated inflammatory conditions requires more *in vitro* and clinical experiments to demonstrate.

## Conclusion

In this study, a well-fitting nomogram model was constructed to predict the incidence of osteomyelitis based on six significant m6A regulators using a random forest approach, while two m6A isoforms were obtained using unsupervised clustering analysis. Furthermore, METTL3 and LRPPRC were determined to be closely associated with immune infiltration by immune infiltration analysis, which will provide guidance for personalized immunotherapy of osteomyelitis patients in the future.

## Data Availability

The original contributions presented in the study are included in the article/Supplementary Material, further inquiries can be directed to the corresponding authors.

## References

[B1] Abinash M. J. Vasudevan V. (2022). Boundaries tuned support vector machine (BT-SVM) classifier for cancer prediction from gene selection. Comput. Methods Biomech. Biomed. Engin. 25 (7), 794–807. 10.1080/10255842.2021.1981300 34585639

[B2] Berendt A. R. Peters E. J. Bakker K. Embil J. M. Eneroth M. Hinchliffe R. J. (2008). Diabetic foot osteomyelitis: A progress report on diagnosis and a systematic review of treatment. Diabetes. Metab. Res. Rev. 24 (1), S145–S161. 10.1002/dmrr.836 18442163

[B3] Boulias K. Toczydłowska-Socha D. Hawley B. R. Liberman N. Takashima K. Zaccara S. (2019). Identification of the m(6)Am methyltransferase PCIF1 reveals the location and functions of m(6)Am in the transcriptome. Mol. Cell 75 (3), 631–643. e8. 10.1016/j.molcel.2019.06.006 31279658 PMC6703822

[B4] Brière G. Darbo É. Thébault P. Uricaru R. (2021). Consensus clustering applied to multi-omics disease subtyping. BMC Bioinforma. 22 (1), 361. 10.1186/s12859-021-04279-1 PMC8259015 34229612

[B5] Chokkalla A. K. Mehta S. L. Kim T. Chelluboina B. Kim J. Vemuganti R. (2019). Transient focal ischemia significantly alters the m(6)A epitranscriptomic tagging of RNAs in the brain. Stroke 50 (10), 2912–2921. 10.1161/STROKEAHA.119.026433 31436138 PMC6759411

[B6] Dai B. Sun F. Cai X. Li C. Liu H. Shang Y. (2021). Significance of RNA N6-methyladenosine regulators in the diagnosis and subtype classification of childhood asthma using the gene expression omnibus database. Front. Genet. 12, 634162. 10.3389/fgene.2021.634162 33763115 PMC7982807

[B7] David C. C. Jacobs D. J. (2014). Principal component analysis: A method for determining the essential dynamics of proteins. Methods Mol. Biol. 1084, 193–226. 10.1007/978-1-62703-658-0_11 24061923 PMC4676806

[B8] Forlino A. Marini J. C. (2016). Osteogenesis imperfecta. Lancet 387 (10028), 1657–1671. 10.1016/S0140-6736(15)00728-X 26542481 PMC7384887

[B9] Hesser C. R. Karijolich J. Dominissini D. He C. Glaunsinger B. A. (2018). N6-methyladenosine modification and the YTHDF2 reader protein play cell type specific roles in lytic viral gene expression during Kaposi's sarcoma-associated herpesvirus infection. PLoS Pathog. 14 (4), e1006995. 10.1371/journal.ppat.1006995 29659627 PMC5919695

[B10] Hogan A. Heppert V. G. Suda A. J. (2013). Arch. Orthop. Trauma Surg. 133 (9), 1183–1196. 10.1007/s00402-013-1785-7 23771127

[B11] Huang S. Cai N. Pacheco P. P. Narrandes S. Wang Y. Xu W. (2018). Applications of support vector machine (SVM) learning in cancer Genomics. Cancer Genomics Proteomics 15 (1), 41–51. 10.21873/cgp.20063 29275361 PMC5822181

[B12] Huang W. Chen T. Q. Fang K. Zeng Z. C. Ye H. Chen Y. Q. (2021). N6-methyladenosine methyltransferases: Functions, regulation, and clinical potential. J. Hematol. Oncol. 14 (1), 103. 10.1186/s13045-019-0800-z 34315512 PMC8313886

[B13] Iasonos A. Schrag D. Raj G. V. Panageas K. S. (2008). How to build and interpret a nomogram for cancer prognosis. J. Clin. Oncol. 26 (8), 1364–1370. 10.1200/JCO.2007.12.9791 18323559

[B14] Kanehisa M. Goto S. Sato Y. Furumichi M. Tanabe M. (2012). KEGG for integration and interpretation of large-scale molecular data sets. Nucleic Acids Res. 40, D109–D114. 10.1093/nar/gkr988 22080510 PMC3245020

[B15] Kong Y. Zhang Y. Cai Y. Li D. Yi B. Xu Q. (2022). METTL3 mediates osteoblast apoptosis by regulating endoplasmic reticulum stress during LPS-induced inflammation. Cell. Signal. 95, 110335. 10.1016/j.cellsig.2022.110335 35461899

[B16] Larsen M. J. Thomassen M. Tan Q. Sørensen K. P. Kruse T. A. (2014). Microarray-based RNA profiling of breast cancer: Batch effect removal improves cross-platform consistency. Biomed. Res. Int. 2014, 651751. 10.1155/2014/651751 25101291 PMC4101981

[B17] Li F. Zhang P. (2021). The N6-methyladenosine- (m6A-) associated genes act as strong key biomarkers for the prognosis of pancreatic adenocarcinoma. Comput. Math. Methods Med. 2021, 8715823. 10.1155/2021/8715823 34840598 PMC8626177

[B18] Linder B. Grozhik A. V. Olarerin-George A. O. Meydan C. Mason C. E. Jaffrey S. R. (2015). Single-nucleotide-resolution mapping of m6A and m6Am throughout the transcriptome. Nat. Methods 12 (8), 767–772. 10.1038/nmeth.3453 26121403 PMC4487409

[B19] Liu C. Yang Z. Li R. Wu Y. Chi M. Gao S. (2021). Potential roles of N6-methyladenosine (m6A) in immune cells. J. Transl. Med. 19 (1), 251. 10.1186/s12967-021-02918-y 34103054 PMC8186046

[B20] Liu L. Li H. Hu D. Wang Y. Shao W. Zhong J. (2022). Insights into N6-methyladenosine and programmed cell death in cancer. Mol. Cancer 21 (1), 32. 10.1186/s12943-022-01508-w 35090469 PMC8796496

[B21] Liu T. Wei Q. Jin J. Luo Q. Liu Y. Yang Y. (2020). The m6A reader YTHDF1 promotes ovarian cancer progression via augmenting EIF3C translation. Nucleic Acids Res. 48 (7), 3816–3831. 10.1093/nar/gkaa048 31996915 PMC7144925

[B22] Liu Y. Liu Z. Tang H. Shen Y. Gong Z. Xie N. (2019). The N(6)-methyladenosine (m(6)A)-forming enzyme METTL3 facilitates M1 macrophage polarization through the methylation of STAT1 mRNA. Am. J. Physiol. Cell Physiol. 317 (4), C762–c75. 10.1152/ajpcell.00212.2019 31365297

[B23] Livak K. J. Schmittgen T. D. (2001). Analysis of relative gene expression data using real-time quantitative PCR and the 2(-Delta Delta C(T)) Method. Methods 25 (4), 402–408. 10.1006/meth.2001.1262 11846609

[B24] Luo J. Xu T. Sun K. (2021). N6-Methyladenosine RNA modification in inflammation: Roles, mechanisms, and applications. Front. Cell Dev. Biol. 9, 670711. 10.3389/fcell.2021.670711 34150765 PMC8213350

[B25] Ma X. Xia W. Zong Y. Jiang C. Shan H. Lin Y. (2021). Tumor necrosis factor-α promotes Staphylococcus aureus-induced osteomyelitis through downregulating endothelial nitric oxide synthase. J. Microbiol. Immunol. Infect. 54 (6), 1018–1027. 10.1016/j.jmii.2020.08.002 32861626

[B26] Ma Z. Gao X. Shuai Y. Xing X. Ji J. (2021). The m6A epitranscriptome opens a new charter in immune system logic. Epigenetics 16 (8), 819–837. 10.1080/15592294.2020.1827722 33070685 PMC8331015

[B27] Marini J. C. Blissett A. R. (2013). New genes in bone development: what's new in osteogenesis imperfecta. J. Clin. Endocrinol. Metab. 98 (8), 3095–3103. 10.1210/jc.2013-1505 23771926 PMC3733862

[B28] Müller C. Schillert A. Röthemeier C. Trégouët D. A. Proust C. Binder H. (2016). Removing batch effects from longitudinal gene expression - quantile normalization plus ComBat as best approach for microarray transcriptome data. PLoS One 11 (6), e0156594. 10.1371/journal.pone.0156594 27272489 PMC4896498

[B29] Myllyharju J. Kivirikko K. I. (2001). Collagens and collagen-related diseases. Ann. Med. 33 (1), 7–21. 10.3109/07853890109002055 11310942

[B30] Noskin G. A. Rubin R. J. Schentag J. J. Kluytmans J. Hedblom E. C. Smulders M. (2005). The burden of *Staphylococcus aureus* infections on hospitals in the United States: An analysis of the 2000 and 2001 nationwide inpatient sample database. Arch. Intern. Med. 165 (15), 1756–1761. 10.1001/archinte.165.15.1756 16087824

[B31] Pasquet J. Chevalier Y. Couval E. Bouvier D. Bolzinger M. A. (2015). Zinc oxide as a new antimicrobial preservative of topical products: Interactions with common formulation ingredients. Int. J. Pharm. 479 (1), 88–95. 10.1016/j.ijpharm.2014.12.031 25527211

[B32] Peltola H. Pääkkönen M. (2014). Acute osteomyelitis in children. N. Engl. J. Med. 370 (4), 1365–1366. 10.1056/NEJMc1402234 24693913

[B33] Polan D. F. Brady S. L. Kaufman R. A. (2016). Tissue segmentation of computed tomography images using a random forest algorithm: A feasibility study. Phys. Med. Biol. 61 (17), 6553–6569. 10.1088/0031-9155/61/17/6553 27530679 PMC5039942

[B34] Ramos-Vara J. A. (2005). Technical aspects of immunohistochemistry. Vet. Pathol. 42 (4), 405–426. 10.1354/vp.42-4-405 16006601

[B35] Ritchie M. E. Phipson B. Wu D. Hu Y. Law C. W. Shi W. (2015). Limma powers differential expression analyses for RNA-sequencing and microarray studies. Nucleic Acids Res. 43 (7), e47. 10.1093/nar/gkv007 25605792 PMC4402510

[B36] Scott R. J. Christofersen M. R. Robertson W. W. Jr. Davidson R. S. Rankin L. Drummond D. S. (1990). Acute osteomyelitis in children: A review of 116 cases. J. Pediatr. Orthop. 10 (5), 649–652. 10.1097/01241398-199009000-00015 2203820

[B37] Seiler M. Huang C. C. Szalma S. Bhanot G. (2010). ConsensusCluster: A software tool for unsupervised cluster discovery in numerical data. Omics 14 (1), 109–113. 10.1089/omi.2009.0083 20141333

[B38] Shi H. Wei J. He C. (2019). Where, when, and how: Context-dependent functions of RNA methylation writers, readers, and erasers. Mol. Cell 74 (4), 640–650. 10.1016/j.molcel.2019.04.025 31100245 PMC6527355

[B39] Shriwas O. Mohapatra P. Mohanty S. Dash R. (2020). The impact of m6A RNA modification in therapy resistance of cancer: Implication in chemotherapy, radiotherapy, and immunotherapy. Front. Oncol. 10, 612337. 10.3389/fonc.2020.612337 33718113 PMC7947626

[B40] The gene Ontology (GO) project in 2006. Nucleic Acids Res. 2006;34:D322–D326. 10.1093/nar/gkj021 16381878 PMC1347384

[B41] Unkila-Kallio L. Kallio M. J. Eskola J. Peltola H. (1994). Serum C-reactive protein, erythrocyte sedimentation rate, and white blood cell count in acute hematogenous osteomyelitis of children. Pediatrics 93 (1), 59–62. 10.1542/peds.93.1.59 8265325

[B42] Vergidis P. Schmidt-Malan S. M. Mandrekar J. N. Steckelberg J. M. Patel R. (2015). Comparative activities of vancomycin, tigecycline and rifampin in a rat model of methicillin-resistant *Staphylococcus aureus* osteomyelitis. J. Infect. 70 (6), 609–615. 10.1016/j.jinf.2014.12.016 25576292

[B43] Wang H. Hu X. Huang M. Liu J. Gu Y. Ma L. (2019). Mettl3-mediated mRNA m(6)A methylation promotes dendritic cell activation. Nat. Commun. 10 (1), 1898. 10.1038/s41467-019-09903-6 31015515 PMC6478715

[B44] Wang J. Yan S. Lu H. Wang S. Xu D. (2019). METTL3 attenuates LPS-induced inflammatory response in macrophages via NF-κB signaling pathway. Mediat. Inflamm. 2019, 3120391. 10.1155/2019/3120391 PMC6854952 31772500

[B45] Wang T. Kong S. Tao M. Ju S. (2020). The potential role of RNA N6-methyladenosine in Cancer progression. Mol. Cancer 19 (1), 88. 10.1186/s12943-020-01204-7 32398132 PMC7216508

[B46] Wilkerson M. D. Hayes D. N. (2010). ConsensusClusterPlus: A class discovery tool with confidence assessments and item tracking. Bioinformatics 26 (12), 1572–1573. 10.1093/bioinformatics/btq170 20427518 PMC2881355

[B47] Xiao B. Liu L. Li A. Xiang C. Wang P. Li H. (2020). Identification and verification of immune-related gene prognostic signature based on ssGSEA for osteosarcoma. Front. Oncol. 10, 607622. 10.3389/fonc.2020.607622 33384961 PMC7771722

[B48] Xiao W. Adhikari S. Dahal U. Chen Y. S. Hao Y. J. Sun B. F. (2016). Nuclear m(6)A reader YTHDC1 regulates mRNA splicing. Mol. Cell 61 (4), 507–519. 10.1016/j.molcel.2016.01.012 26876937

[B49] Yu G. Wang L. G. Han Y. He Q. Y. (2012). clusterProfiler: an R package for comparing biological themes among gene clusters. Omics 16 (5), 284–287. 10.1089/omi.2011.0118 22455463 PMC3339379

[B50] Yu R. Li Q. Feng Z. Cai L. Xu Q. (2019). m6A reader YTHDF2 regulates LPS-induced inflammatory response. Int. J. Mol. Sci. 20 (6), E1323. 10.3390/ijms20061323 PMC6470741 30875984

[B51] Zalavras C. G. (2017). Prevention of infection in open fractures. Infect. Dis. Clin. North Am. 31 (2), 339–352. 10.1016/j.idc.2017.01.005 28292542

[B52] Zhang B. Wu Q. Li B. Wang D. Wang L. Zhou Y. L. (2020). m(6 A regulator-mediated methylation modification patterns and tumor microenvironment infiltration characterization in gastric cancer. Mol. Cancer 19 (1), 53. 10.1186/s12943-020-01170-0 32164750 PMC7066851

[B53] Zhang H. Meltzer P. Davis S. (2013). RCircos: an R package for Circos 2D track plots. BMC Bioinforma. 14, 244. 10.1186/1471-2105-14-244 PMC3765848 23937229

[B54] Zhang X. Zhang S. Yan X. Shan Y. Liu L. Zhou J. (2021). m6A regulator-mediated RNA methylation modification patterns are involved in immune microenvironment regulation of periodontitis. J. Cell. Mol. Med. 25 (7), 3634–3645. 10.1111/jcmm.16469 33724691 PMC8034465

[B55] Zhang Y. Gu X. Li D. Cai L. Xu Q. (2019). METTL3 regulates osteoblast differentiation and inflammatory response via Smad signaling and MAPK signaling. Int. J. Mol. Sci. 21 (1), E199. 10.3390/ijms21010199 PMC6981640 31892163

[B56] Zhang Y. Parmigiani G. Johnson W. E. (2020). ComBat-seq: Batch effect adjustment for RNA-seq count data. Nar. Genom. Bioinform. 2 (3), lqaa078. 10.1093/nargab/lqaa078 33015620 PMC7518324

[B57] Zhao H. Xu Y. Xie Y. Zhang L. Gao M. Li S. (2021). m6A regulators is differently expressed and correlated with immune response of esophageal cancer. Front. Cell Dev. Biol. 9, 650023. 10.3389/fcell.2021.650023 33748145 PMC7970005

[B58] Zhao S. Guo Y. Sheng Q. Shyr Y. (2014). Advanced heat map and clustering analysis using heatmap3. Biomed. Res. Int. 2014, 986048. 10.1155/2014/986048 25143956 PMC4124803

[B59] Zhao W. Qi X. Liu L. Ma S. Liu J. Wu J. (2020). Epigenetic regulation of m(6)A modifications in human cancer. Mol. Ther. Nucleic Acids 19, 405–412. 10.1016/j.omtn.2019.11.022 31887551 PMC6938965

[B60] Zhao X. Ge L. Wang J. Song Z. Ni B. He X. (2021). Exploration of potential integrated models of N6-methyladenosine immunity in systemic lupus erythematosus by bioinformatic analyses. Front. Immunol. 12, 752736. 10.3389/fimmu.2021.752736 35197962 PMC8859446

[B61] Zhu Z. M. Huo F. C. Pei D. S. (2020). Function and evolution of RNA N6-methyladenosine modification. Int. J. Biol. Sci. 16 (11), 1929–1940. 10.7150/ijbs.45231 32398960 PMC7211178

[B62] Zong X. Zhao J. Wang H. Lu Z. Wang F. Du H. (2019). Mettl3 deficiency sustains long-chain fatty acid absorption through suppressing traf6-dependent inflammation response. J. Immunol. 202 (2), 567–578. 10.4049/jimmunol.1801151 30567729 PMC6321842

