# Supplementary material for: Diagnostic signature, subtype classification, and immune infiltration of key m6A regulators in osteomyelitis patients
Source: Front Genet. 2022 Dec 5;13:1044264. doi: 10.3389/fgene.2022.1044264 (PMC9760713; doi:10.3389/fgene.2022.1044264)
Supplement: Supplementary file 1 [file DataSheet1.ZIP › Supplemental Table 1.docx]

| Supplemental Table 1: The primer used for 6 m6A regulators and GAPDH. | | |
| --- | --- | --- |
| Targeted gene | Forward (5′-3′) | Reverse (3′-5′) |
| METTL3 | TGGATTGCGATGTGATTGT | CTGGTTTGTGACTGGTGGA |
| RBM15B | CCCTCCGCACCTTCTGTATT | CCTCCGCTTTCGTCTCTCTT |
| CBLL1 | CGCTCCGCACAAGCCGAGTG | GCTCGAAGGCCACTGTTGTT |
| YTHDC1 | AACTGATTTCTAAGCCACTA | CATCTCCATCTTCATCTACC |
| YTHDF2 | CCTCTATTGGCTTTTCCTAT | CTCTCCGTTGCTCAGTTGTC |
| LRPPRC | CGCTGCCTATTGTAGTGTTG | ATTGGAAGGTCCCTCGTTTT |
| GAPDH | GACCCCTTCATTGACCTCAAC | GCCATCACGCCACAGCTTTCC |

| Supplemental Table 2: The m6A score of each sample with osteomyelitis. | |
| --- | --- |
| Sample ID | m6A score |
| GSM403285 control | 0.422918937 |
| GSM403305 control | -0.113248494 |
| GSM403313 control | 1.441621856 |
| GSM403323 control | 2.119345508 |
| GSM403327 control | 1.735568578 |
| GSM403329 control | 2.926501434 |
| GSM403331 control | 1.958978108 |
| GSM403412 control | 1.669382211 |
| GSM403547 control | 1.198789905 |
| GSM403552 control | 0.965574519 |
| GSM403555 control | 1.759951474 |
| GSM403562 control | -1.376041326 |
| GSM403563 control | 0.914711476 |
| GSM403564 control | 0.656595749 |
| GSM403566 control | 0.255861738 |
| GSM403567 control | 0.480440421 |
| GSM403568 control | 0.084673959 |
| GSM403569 control | -2.0641123 |
| GSM403570 control | -0.412171229 |
| GSM403284 control | 1.818919917 |
| GSM403304 control | 1.88272347 |
| GSM403312 control | 0.978596072 |
| GSM403322 control | 0.568225647 |
| GSM403326 control | 0.565003582 |
| GSM403328 control | 0.768407131 |
| GSM403330 control | -0.933102264 |
| GSM403411 control | 2.058670839 |
| GSM403546 control | 0.963600834 |
| GSM403551 control | 2.304043345 |
| GSM745660 control | -1.637725195 |
| GSM745661 control | -0.688595282 |
| GSM745662 control | -1.167945879 |
| GSM745663 control | 0.877515917 |
| GSM745664 control | 0.555206606 |
| GSM745665 control | 2.182525175 |
| GSM745666 control | 0.172377889 |
| GSM745667 control | 2.181538639 |
| GSM745668 control | 0.124681842 |
| GSM745669 control | 1.636300717 |
| GSM745670 control | 1.563937501 |
| GSM745671 control | 0.841293785 |
| GSM745672 control | 2.147455216 |
| GSM745673 control | 1.066807592 |
| GSM745674 control | 1.941372767 |
| GSM745675 control | 0.15696124 |
| GSM745676 control | -0.139663092 |
| GSM745677 control | 0.573765586 |
| GSM745678 control | -0.026131092 |
| GSM745679 control | 0.336091517 |
| GSM745680 control | -0.722708729 |
| GSM745681 control | 3.051156695 |
| GSM745722 control | 2.066213808 |
| GSM745723 control | 0.802680869 |
| GSM745724 control | 0.912884702 |
| GSM745725 control | 1.925203342 |
| GSM745726 control | 0.037163298 |
| GSM745727 control | 2.914100614 |
| GSM745728 control | 1.864612118 |
| GSM745729 control | 2.016836934 |
| GSM745730 control | 3.412872819 |
| GSM745731 control | 2.77198991 |
| GSM745732 control | -0.174419292 |
| GSM745733 control | 1.665272883 |
| GSM745734 control | -0.055362352 |
| GSM745735 control | 0.075725655 |
| GSM745736 control | 0.673815864 |
| GSM745737 control | 0.104900276 |
| GSM745738 control | 2.005031563 |
| GSM745739 control | 0.318169675 |
| GSM745740 control | 1.553513562 |
| GSM745741 control | 1.237592803 |
| GSM745742 control | -0.394060796 |
| GSM745743 control | 0.603023918 |
| GSM403283 treatment | -2.250583712 |
| GSM403287 treatment | -3.485185595 |
| GSM403289 treatment | -2.40518514 |
| GSM403291 treatment | -0.42932194 |
| GSM403293 treatment | 0.832521892 |
| GSM403295 treatment | 1.930045544 |
| GSM403297 treatment | 1.005179385 |
| GSM403299 treatment | -0.200642066 |
| GSM403301 treatment | 2.21617092 |
| GSM403303 treatment | 0.801426027 |
| GSM403307 treatment | -1.211331708 |
| GSM403309 treatment | -0.792100898 |
| GSM403311 treatment | 0.345763296 |
| GSM403315 treatment | -0.195117437 |
| GSM403317 treatment | 0.635355313 |
| GSM403319 treatment | 0.598398275 |
| GSM403321 treatment | 2.113012784 |
| GSM403325 treatment | -1.125820094 |
| GSM403394 treatment | 0.556061357 |
| GSM403396 treatment | -2.904177039 |
| GSM403398 treatment | 0.104704467 |
| GSM403400 treatment | -1.622934214 |
| GSM403402 treatment | -1.423508941 |
| GSM403404 treatment | -2.595984641 |
| GSM403406 treatment | -0.451090131 |
| GSM403408 treatment | -0.254001419 |
| GSM403410 treatment | -0.491840924 |
| GSM403414 treatment | -1.240294833 |
| GSM403416 treatment | 1.08062261 |
| GSM403418 treatment | 1.37005193 |
| GSM403420 treatment | -0.279546273 |
| GSM403422 treatment | -1.169835543 |
| GSM403425 treatment | 1.021536311 |
| GSM403428 treatment | 0.078244391 |
| GSM403430 treatment | -1.497018934 |
| GSM403434 treatment | -0.392490485 |
| GSM403436 treatment | -0.835413518 |
| GSM403438 treatment | -2.354362658 |
| GSM403440 treatment | -1.619845517 |
| GSM403442 treatment | 0.573086745 |
| GSM403549 treatment | 0.139036401 |
| GSM403554 treatment | -0.062696471 |
| GSM403550 treatment | 2.87856513 |
| GSM403560 treatment | -1.764611942 |
| GSM403561 treatment | 2.184438904 |
| GSM403571 treatment | 0.282568904 |
| GSM403572 treatment | 2.287755241 |
| GSM403573 treatment | -3.019962215 |
| GSM403574 treatment | -0.477854735 |
| GSM403575 treatment | -2.221623124 |
| GSM403576 treatment | 1.292230014 |
| GSM403282 treatment | -0.337030038 |
| GSM403286 treatment | -1.27389311 |
| GSM403288 treatment | 0.881483364 |
| GSM403290 treatment | -2.892858497 |
| GSM403292 treatment | -2.277708069 |
| GSM403294 treatment | -1.007905385 |
| GSM403296 treatment | 0.801094974 |
| GSM403298 treatment | -3.954645953 |
| GSM403300 treatment | 0.872888243 |
| GSM403302 treatment | 1.107014372 |
| GSM403306 treatment | 1.28780546 |
| GSM403308 treatment | -0.352323202 |
| GSM403310 treatment | -0.665170693 |
| GSM403314 treatment | -0.361330353 |
| GSM403316 treatment | 0.636048276 |
| GSM403318 treatment | -1.646201506 |
| GSM403320 treatment | -0.018638273 |
| GSM403324 treatment | 2.943474144 |
| GSM403393 treatment | -1.187714268 |
| GSM403395 treatment | -0.320087475 |
| GSM403397 treatment | -1.177005117 |
| GSM403399 treatment | -0.056236673 |
| GSM403401 treatment | -3.025352682 |
| GSM403403 treatment | -3.472226869 |
| GSM403405 treatment | 0.186511873 |
| GSM403407 treatment | -0.685587736 |
| GSM403409 treatment | -0.743717035 |
| GSM403413 treatment | 1.207307142 |
| GSM403415 treatment | -0.993169946 |
| GSM403417 treatment | -1.586730688 |
| GSM403419 treatment | -1.876455994 |
| GSM403421 treatment | 0.511361563 |
| GSM403423 treatment | 2.169141369 |
| GSM403424 treatment | 3.203422826 |
| GSM403426 treatment | -2.83872506 |
| GSM403427 treatment | -1.293571797 |
| GSM403429 treatment | -2.056410532 |
| GSM403431 treatment | -2.161826605 |
| GSM403432 treatment | -3.224442017 |
| GSM403433 treatment | -2.004511107 |
| GSM403435 treatment | 4.117793193 |
| GSM403437 treatment | 4.416376971 |
| GSM403439 treatment | 0.341608717 |
| GSM403441 treatment | 0.701615005 |
| GSM403548 treatment | 3.070676164 |
| GSM403553 treatment | 3.989825734 |
| GSM745689 treatment | 0.323016784 |
| GSM745691 treatment | -1.080522457 |
| GSM745774 treatment | -0.430039061 |
| GSM745716 treatment | -2.390790156 |
| GSM745802 treatment | -2.743660429 |
| GSM745748 treatment | -2.080514897 |
| GSM745749 treatment | -2.587565996 |
| GSM745775 treatment | -0.139382669 |
| GSM745773 treatment | -2.72312863 |
| GSM745766 treatment | 0.606487861 |
| GSM745699 treatment | -2.212472397 |
| GSM745700 treatment | -2.070936784 |
| GSM745701 treatment | -0.654810723 |
| GSM745708 treatment | -0.481915297 |
| GSM745715 treatment | -0.511411318 |
| GSM745693 treatment | 1.117719667 |
| GSM745709 treatment | -0.24971988 |
| GSM745710 treatment | -1.573475718 |
| GSM745692 treatment | -1.244667353 |
| GSM745685 treatment | -0.003583148 |
| GSM745695 treatment | -0.426537247 |
| GSM745694 treatment | -0.384749736 |
| GSM745718 treatment | 0.007210317 |
| GSM745797 treatment | -2.451340436 |
| GSM745793 treatment | -1.697061393 |
| GSM745790 treatment | -0.642407664 |
| GSM745788 treatment | -1.691005589 |
| GSM745776 treatment | -0.512111583 |
| GSM745771 treatment | 1.489136772 |
| GSM745706 treatment | -2.917085238 |
| GSM745768 treatment | 1.000071559 |
| GSM745687 treatment | 1.387426764 |
| GSM745721 treatment | -2.31766258 |
| GSM745684 treatment | -3.222945919 |
| GSM745707 treatment | -1.093283697 |
| GSM745714 treatment | -1.65900592 |
| GSM745698 treatment | -0.727400964 |
| GSM745755 treatment | -1.921570234 |
| GSM745770 treatment | -2.5590837 |
