# Supplementary material for: Diagnostic signature, subtype classification, and immune infiltration of key m6A regulators in osteomyelitis patients
Source: Front Genet. 2022 Dec 5;13:1044264. doi: 10.3389/fgene.2022.1044264 (PMC9760713; doi:10.3389/fgene.2022.1044264)
Supplement: Supplementary file 1 [file DataSheet1.ZIP › Supplemental Table 2.docx]

| Supplemental Table 2: The m6A score of each sample with osteomyelitis. | |
| --- | --- |
| Sample ID | m6A score |
| GSM403283 treatment | -2.250583712 |
| GSM403287 treatment | -3.485185595 |
| GSM403289 treatment | -2.40518514 |
| GSM403291 treatment | -0.42932194 |
| GSM403293 treatment | 0.832521892 |
| GSM403295 treatment | 1.930045544 |
| GSM403297 treatment | 1.005179385 |
| GSM403299 treatment | -0.200642066 |
| GSM403301 treatment | 2.21617092 |
| GSM403303 treatment | 0.801426027 |
| GSM403307 treatment | -1.211331708 |
| GSM403309 treatment | -0.792100898 |
| GSM403311 treatment | 0.345763296 |
| GSM403315 treatment | -0.195117437 |
| GSM403317 treatment | 0.635355313 |
| GSM403319 treatment | 0.598398275 |
| GSM403321 treatment | 2.113012784 |
| GSM403325 treatment | -1.125820094 |
| GSM403394 treatment | 0.556061357 |
| GSM403396 treatment | -2.904177039 |
| GSM403398 treatment | 0.104704467 |
| GSM403400 treatment | -1.622934214 |
| GSM403402 treatment | -1.423508941 |
| GSM403404 treatment | -2.595984641 |
| GSM403406 treatment | -0.451090131 |
| GSM403408 treatment | -0.254001419 |
| GSM403410 treatment | -0.491840924 |
| GSM403414 treatment | -1.240294833 |
| GSM403416 treatment | 1.08062261 |
| GSM403418 treatment | 1.37005193 |
| GSM403420 treatment | -0.279546273 |
| GSM403422 treatment | -1.169835543 |
| GSM403425 treatment | 1.021536311 |
| GSM403428 treatment | 0.078244391 |
| GSM403430 treatment | -1.497018934 |
| GSM403434 treatment | -0.392490485 |
| GSM403436 treatment | -0.835413518 |
| GSM403438 treatment | -2.354362658 |
| GSM403440 treatment | -1.619845517 |
| GSM403442 treatment | 0.573086745 |
| GSM403549 treatment | 0.139036401 |
| GSM403554 treatment | -0.062696471 |
| GSM403550 treatment | 2.87856513 |
| GSM403560 treatment | -1.764611942 |
| GSM403561 treatment | 2.184438904 |
| GSM403571 treatment | 0.282568904 |
| GSM403572 treatment | 2.287755241 |
| GSM403573 treatment | -3.019962215 |
| GSM403574 treatment | -0.477854735 |
| GSM403575 treatment | -2.221623124 |
| GSM403576 treatment | 1.292230014 |
| GSM403282 treatment | -0.337030038 |
| GSM403286 treatment | -1.27389311 |
| GSM403288 treatment | 0.881483364 |
| GSM403290 treatment | -2.892858497 |
| GSM403292 treatment | -2.277708069 |
| GSM403294 treatment | -1.007905385 |
| GSM403296 treatment | 0.801094974 |
| GSM403298 treatment | -3.954645953 |
| GSM403300 treatment | 0.872888243 |
| GSM403302 treatment | 1.107014372 |
| GSM403306 treatment | 1.28780546 |
| GSM403308 treatment | -0.352323202 |
| GSM403310 treatment | -0.665170693 |
| GSM403314 treatment | -0.361330353 |
| GSM403316 treatment | 0.636048276 |
| GSM403318 treatment | -1.646201506 |
| GSM403320 treatment | -0.018638273 |
| GSM403324 treatment | 2.943474144 |
| GSM403393 treatment | -1.187714268 |
| GSM403395 treatment | -0.320087475 |
| GSM403397 treatment | -1.177005117 |
| GSM403399 treatment | -0.056236673 |
| GSM403401 treatment | -3.025352682 |
| GSM403403 treatment | -3.472226869 |
| GSM403405 treatment | 0.186511873 |
| GSM403407 treatment | -0.685587736 |
| GSM403409 treatment | -0.743717035 |
| GSM403413 treatment | 1.207307142 |
| GSM403415 treatment | -0.993169946 |
| GSM403417 treatment | -1.586730688 |
| GSM403419 treatment | -1.876455994 |
| GSM403421 treatment | 0.511361563 |
| GSM403423 treatment | 2.169141369 |
| GSM403424 treatment | 3.203422826 |
| GSM403426 treatment | -2.83872506 |
| GSM403427 treatment | -1.293571797 |
| GSM403429 treatment | -2.056410532 |
| GSM403431 treatment | -2.161826605 |
| GSM403432 treatment | -3.224442017 |
| GSM403433 treatment | -2.004511107 |
| GSM403435 treatment | 4.117793193 |
| GSM403437 treatment | 4.416376971 |
| GSM403439 treatment | 0.341608717 |
| GSM403441 treatment | 0.701615005 |
| GSM403548 treatment | 3.070676164 |
| GSM403553 treatment | 3.989825734 |
| GSM745689 treatment | 0.323016784 |
| GSM745691 treatment | -1.080522457 |
| GSM745774 treatment | -0.430039061 |
| GSM745716 treatment | -2.390790156 |
| GSM745802 treatment | -2.743660429 |
| GSM745748 treatment | -2.080514897 |
| GSM745749 treatment | -2.587565996 |
| GSM745775 treatment | -0.139382669 |
| GSM745773 treatment | -2.72312863 |
| GSM745766 treatment | 0.606487861 |
| GSM745699 treatment | -2.212472397 |
| GSM745700 treatment | -2.070936784 |
| GSM745701 treatment | -0.654810723 |
| GSM745708 treatment | -0.481915297 |
| GSM745715 treatment | -0.511411318 |
| GSM745693 treatment | 1.117719667 |
| GSM745709 treatment | -0.24971988 |
| GSM745710 treatment | -1.573475718 |
| GSM745692 treatment | -1.244667353 |
| GSM745685 treatment | -0.003583148 |
| GSM745695 treatment | -0.426537247 |
| GSM745694 treatment | -0.384749736 |
| GSM745718 treatment | 0.007210317 |
| GSM745797 treatment | -2.451340436 |
| GSM745793 treatment | -1.697061393 |
| GSM745790 treatment | -0.642407664 |
| GSM745788 treatment | -1.691005589 |
| GSM745776 treatment | -0.512111583 |
| GSM745771 treatment | 1.489136772 |
| GSM745706 treatment | -2.917085238 |
| GSM745768 treatment | 1.000071559 |
| GSM745687 treatment | 1.387426764 |
| GSM745721 treatment | -2.31766258 |
| GSM745684 treatment | -3.222945919 |
| GSM745707 treatment | -1.093283697 |
| GSM745714 treatment | -1.65900592 |
| GSM745698 treatment | -0.727400964 |
| GSM745755 treatment | -1.921570234 |
| GSM745770 treatment | -2.5590837 |
