# Supplementary material for: Diagnostic signature, subtype classification, and immune infiltration of key m6A regulators in osteomyelitis patients
Source: Front Genet. 2022 Dec 5;13:1044264. doi: 10.3389/fgene.2022.1044264 (PMC9760713; doi:10.3389/fgene.2022.1044264)
Supplement: Supplementary file 1 [file DataSheet1.ZIP › Supplemental Figure S1.docx]

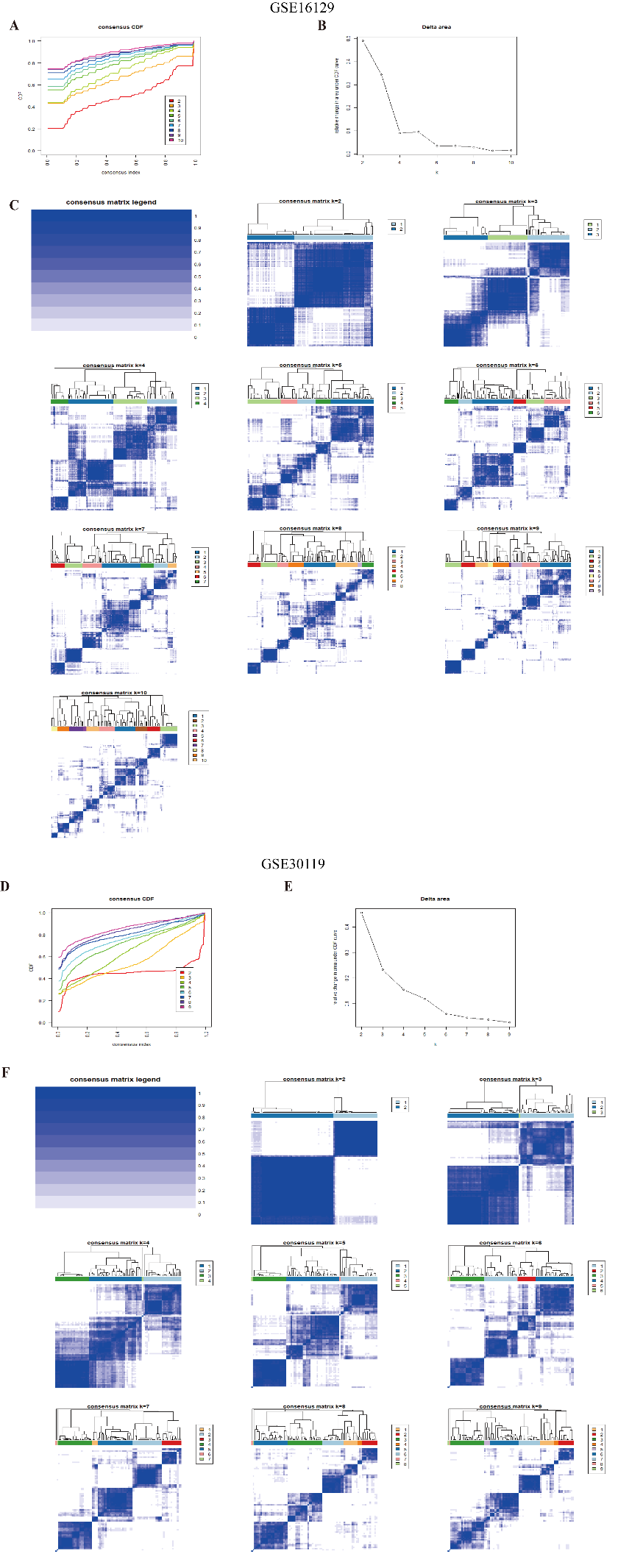


**Supplemental Figure S1**: Results of molecular subtypes based on m6A regulators. (A) The cumulative distribution function (CDF) on m6A subtypes of the test group. (B) The value of K corresponding to when the CDF delta area its approximate maximum is the best grouping result (k=2). (C) Results of different clusters in the test group (k=1-10). (D) The cumulative distribution function (CDF) on m6A subtypes of the validation group. (E) The value of K corresponding to when the CDF delta area its approximate maximum is the best grouping result (k=2). (F) Results of different clusters in the test group (k=1-9).
